# A neonicotinoid pesticide impairs foraging, but not learning, in free-flying bumblebees

**DOI:** 10.1038/s41598-019-39701-5

**Published:** 2019-03-18

**Authors:** F. Muth, A. S. Leonard

**Affiliations:** Department of Biology, University of Nevada, Reno, NV 89557 USA

## Abstract

Neonicotinoids are widely-used pesticides implicated in the decline of bees, known to have sub-lethal effects on bees’ foraging and colony performance. One proposed mechanism for these negative effects is impairment to bees’ ability to learn floral associations. However, the effects of neonicotinoids on learning performance have largely been addressed using a single protocol, where immobilized bees learn an association based on a single sensory modality. We thus have an incomplete understanding of how these pesticides affect bee learning in more naturalistic foraging scenarios. We carried out the first free-foraging study into the effects of acute exposure of a neonicotinoid (imidacloprid) on bumblebees’ (*Bombus impatiens*) ability to learn associations with visual stimuli. We uncovered dose-dependent detrimental effects on motivation to initiate foraging, amount of nectar collected, and initiation of subsequent foraging bouts. However, we did not find any impairment to bees’ ability to learn visual associations. While not precluding the possibility that other forms of learning are impaired, our findings suggest that some of the major effects of acute neonicotinoid exposure on foraging performance may be due to motivational and/or sensory impairments. In light of these findings, we discuss more broadly how pesticide effects on pollinator cognition might be studied.

## Introduction

Bees are one of the primary pollinators of both native plants and agricultural crops, consisting of around 20,000 species worldwide. Many of these species have undergone recent declines, of concern to scientists and farmers alike^1^. Beyond the implications for food costs and security^2^, wild bee populations play a critical role in terrestrial ecosystems, meaning that their decline will likely have multifarious effects on ecological communities and floral diversity^3^. Bees’ declines are thought to be driven by a number of interacting factors, including changing nutritional resources, habitat loss, pathogen exposure^4–6^, and pesticides^6^.

One widely-used group of pesticides of recent concern are neonicotinoids. While three neonicotinoid pesticides (clothianidin, imidacloprid and thiamethoxam) were recently restricted for outdoor use in the EU (EU Press Release, 27/4/2018), neonicotinoids are still widely used in other parts of the world, including the USA and China^7^. After being applied as seed treatments, neonicotinoids occur in trace levels in the nectar and pollen of crop plants^8^, where they may be consumed or brought back to the colonies of social bees. They also dissolve in water and are long-lasting in the environment, and as such have been found at surprisingly high concentrations in the pollen and nectar of wildflowers near treated crops^9^, meaning a number of wild floral visitors are also likely to come into contact with them.

Bumblebees (genus *Bombus*), are a group of ecologically and economically important pollinators, up to one third of which may be in decline^10^. Bumblebees appear to be particularly sensitive to neonicotinoid pesticides^11,12^, which affect both bumblebee colony growth and foraging efficiency^13–17^. There is evidence to suggest that some of the reported negative impacts of neonicotinoids on bee foraging performance may stem from impairment to learning and memory^18^. Neonicotinoids are agonists of nicotinic acetylcholine receptors (nAChRs), widespread in the insect brain and central nervous system^19–21^. Imidacloprid, a commonly-used neonicotinoid, causes sustained activation of Kenyon cell nAChRs in the mushroom bodies^22^, structures in the insect brain associated with learning, memory, and sensory integration^23,24^. However, neonicotinoids also disrupt odour perception at even earlier stages of processing, at least in in honeybees (*Apis mellifera*), via effects on antennal lobe functionality^25^. Olfactory information may thus be already degraded before reaching the mushroom bodies.

Impairment to cognition is particularly pertinent to generalist foragers such as honeybees and bumblebees, who rely on learning and memory to efficiently collect multiple resources from a diversity of flower species^26^. Bees learn associations between many floral cues (e.g. colour, scent) and rewards such as nectar^27^ and pollen^28^. They also learn how to handle flowers effectively^29^, and how to navigate successfully between flowers and their colony^30^. Bees’ ability to learn may in turn affect colony performance and fitness (^31^ but see^32^). Given this reliance on learning during foraging, cognitive impairment means that colonies may be both less productive and die sooner, and that crops may be pollinated with lower efficiency^33^.

A number of studies have investigated the effects of acute or chronic exposure of neonicotinoids on bees’ ability to learn, generally finding that neonicotinoids cause impairment^18^. For example, a single acute exposure to imidacloprid can impair learning in honeybees^34–36^, although another study found no effect^37^. Honeybees exposed to chronic levels of imidacloprid also show impaired olfactory learning^38,39^. While fewer studies have addressed neonicotinoid effects on bumblebee learning, these have had mixed results: thiamethoxam impaired olfactory learning performance both at acute and chronic exposure levels^40^, while clothianidin did not^41^.

**Figure 1:**
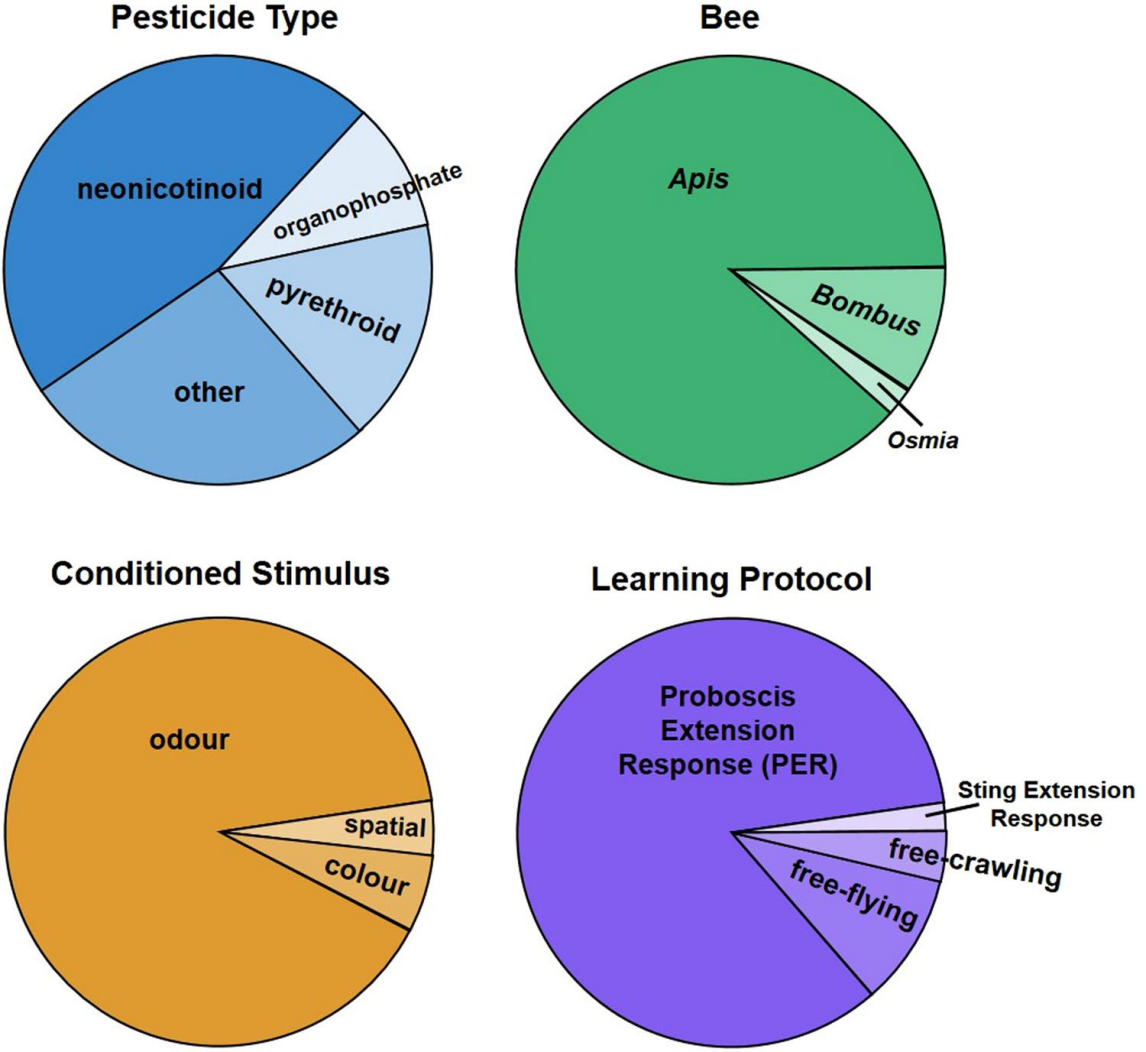
Summary of studies (n = 50) addressing pesticide effects on learning. Studies were identified based on a Web of Science and Google Scholar search of “pesticide bee learning” (June 2018). See Table S1 for details.

One commonality of nearly all studies investigating pesticide effects (both neonicotinoids and other pesticides) on learning in bees is that learning is assessed using olfactory conditioning in the “Proboscis Extension Response” (PER) protocol^42,43^. Indeed, a Web of Science and Google Scholar search of “bee pesticide learning” (June 2018) returned 50 relevant studies; of these, 88% used olfactory conditioning in the PER protocol to measure pesticide effects on cognition (Fig. 1; Table S1). In the PER protocol, bees are immobilized in “harnesses”, allowing only their antennae and proboscis to move. A conditioned stimulus (CS; odour) is then presented with an unconditioned stimulus (US; sucrose) and the bee is conditioned over a series of trials to extend its proboscis for the sucrose reward when being presented with the CS.

The PER protocol has proven very useful in addressing many aspects of bee learning^44^, and there are clear advantages to using a single type of protocol to compare the effects of different types and doses of pesticides (at least in theory: in practice, these papers vary in important parameters such as trial number, inter-trial interval and performance measure used). However, by using a single protocol, we only gain a single perspective on a pesticide’s effects on behaviour. In the case of PER, the protocol’s simplicity comes at the expense of specificity, as performance measures may be overlooked or even misinterpreted. The PER protocol has only been used to address pesticide effects on olfactory conditioning, and indeed more broadly over 90% of the studies on pesticide effects on cognition focus on olfaction (Fig. 1; Table S1). The demonstrated effects of neonicotinoids on olfactory processing^25^ make it difficult to disentangle whether differences in learning performance reflect peripheral or central processes, and thus whether learning that relies on modalities other than olfaction might also be disrupted.

The PER protocol also clearly does not represent the complexity of a natural foraging scenario for bees (as discussed in^45^). Previous work has shown that bees restrained for PER differ from free-moving bees in a number of ways, including accepting different concentrations of sucrose^46^ and toxic substances^47^. Furthermore, more complex effects of pesticides on behaviour may be difficult to discern when using a single simple behavioural measure such as proboscis extension. For example, if neonicotinoids affect bees’ feeding motivation^48,49^, this motivational effect may be difficult to disentangle from a reduction in learning performance: reduced proboscis extension to the conditioned stimulus may be due to a reduction in feeding motivation rather than an impaired ability to learn that the stimulus predicts reward. It is also plausible that being harnessed is itself stressful; stress impacts bee learning and memory (e.g.^50,51^), and might interact with the effects of pesticide exposure in complex ways.

**Figure 2:**
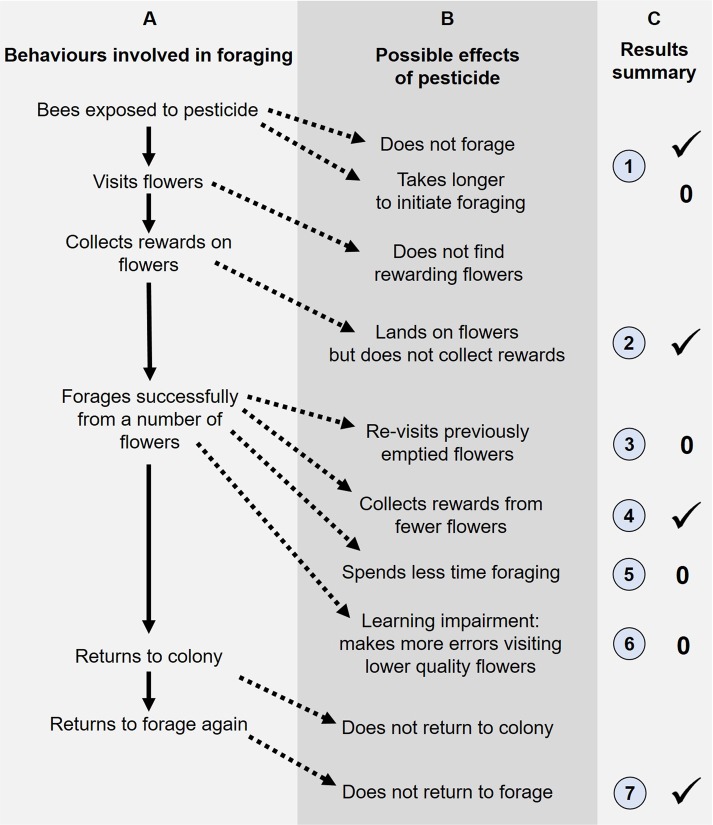
Pesticide exposure may affect bee foraging at a number of stages. Column A lists some of the behaviours that are involved in successful foraging, column B lists the ways these behaviours can be disrupted. Column C lists the behaviours we addressed (numbered as in the Results section); tick symbol = the behaviour was affected in our study; 0 = the behaviour was unaffected.

Given that neonicotinoids may have diverse effects on many aspects of foraging behaviour, a more holistic framework is needed to address effects on behaviour and cognition, which could help inform risk assessment^52^. A given pesticide might affect foraging behaviour at a number of stages (Fig. 2, columns A and B). Impairments at many of these stages could indirectly affect learning performance, without learning *per se* being affected by the direct action of the pesticide. For example, if bees are less motivated to forage, or have motor impairments that limit foraging, they will have less opportunity to learn about flowers. A greater understanding of the specific effects of neonicotinoids on behaviour will help us design appropriate screening assays for pesticides more broadly, designed to test for effects on behaviour that would have the most serious consequences for colony performance^52^. For example, if a pesticide’s main negative effects are on motor abilities, these effects might be more difficult for a colony to compensate for, than olfactory learning (which may be compensated for by the bee relying on visual cues in foraging^53^).

By examining multiple behaviours, we aimed to determine how bumblebee foraging performance might be affected by acute exposure to the neonicotinoid, imidacloprid. We used an experimental approach designed to achieve a balance between ecological realism and experimental control. We allowed individual bumblebee (*Bombus impatiens*) foragers to freely fly between artificial flowers after a known acute pesticide exposure to imidacloprid, across a range of field-realistic doses. We carried out two experiments that varied in the opportunity and motivation for bees to learn. By dissecting out the specific elements of foraging that showed impairment, we discuss factors which should be considered more broadly when examining the effects of any pesticide on pollinator foraging behaviour.

## Methods

### Colony maintenance and selection of subjects

We sequentially tested colonies (Expt. 1 n = 3; Expt. 2 n = 3) of *Bombus impatiens*, purchased with a natal queen and ~70 workers (Koppert Biological Systems MI, USA). These colonies were maintained on ~500 mg honeybee-collected pollen (Koppert Biological Systems MI, USA) provided every 2–3 days, and 30% (w/w) sucrose (details below).

A given colony was connected to a central foraging arena (L x W x H: 122 × 59 × 59 cm), where all training and testing took place, lit from above by an LED light strip (2100 lumens, 4000 K, Lithonia Lighting, Conyers, GA, U.S.A.); the room was illuminated by both fluorescent and natural light. We trained bees to visit artificial flowers (a ‘pre-training array’) in the foraging arena (details in Supplementary Material) and tagged foragers. Because the effects of pesticide exposure may depend on body mass (which is the case at least between species^54^), and since learning ability can vary with body size in *Bombus*^55^, we selected foragers that appeared to be roughly the same size, and then measured them after the experiment to confirm that body size did not differ between treatments (Supplementary Material).

### Training and testing arrays

The artificial flower arrays we used for training and testing consisted of a wooden board (L x W: 60 × 40 cm), painted dark green with acrylic paint (Craftsmart, Irving, TX), with 48 “flowers” (tubes pained green with either human-purple (n = 24) or human-blue (n = 24) circular tops (diameter 3.3 cm); Fig. S1a). Plotted into Chittka’s hexagonal model of bee colour space (Fig. S1;^56^), these stimuli had a chromatic contrast of 0.038, meaning that they are discriminable but relatively difficult to learn to distinguish^57,58^. Flowers also each contained a beige chenille stem (1 cm in length) to imitate an anther, and a sucrose well (outer diameter 7 mm, inner diameter 2.5 mm) containing either 4 µl 50% (w/w) sucrose for rewarding flowers or water (Expt. 1) or 3% NaCl solution (Expt. 2) for unrewarding flowers.

### Pesticide solutions

Pesticide solutions for use in experiments consisted of 93.00 mg of analytical standard imidacloprid powder dissolved in 93 ml of acetone. Aliquots of this solution were then added to 1 kg (889 ml) of 30% (w/w) sucrose solution for use in experiments. For the control solutions containing no pesticide, the same amount of acetone was added to the same volume of 30% (w/w) sucrose solution. Solutions were stored in amber bottles in a refrigerator (at 4 °C), and were always fed to bees immediately after being poured from these bottles (and the solutions then immediately returned to the refrigerator). Fresh solutions were made every 4–5 days. Pesticide solutions were only ever fed to individual bees prior to the experiment (described below) and not offered via any flowers, feeders, or honeypots.

### Dosing bees

To expose subjects to an acute dose of pesticide (experimental treatment) or sucrose (control), we caught a paint-marked forager as it left the colony by allowing it to walk into a black cylindrical container (D × H: 4.5 × 9 cm; Fig. S1b) containing only a small hole at the base. The container was inverted on a plastic surface, and the bee would typically attempt to leave the tube through the small hole (only large enough for its head to emerge). When the bee stuck its head through the hole, we used a pipette to feed it 20 μl of 30% (w/w) sucrose dosed with one of five (Expt. 1) possible concentrations of imidacloprid: 0 (control), 10, 20, 50 or 100 µg/kg. This dose converts to 0, 0.22, 0.45, 1.12, and 2.25 ng/bee or 0, 11.2, 22.5, 56.2 and 112.4 PPB. In Expt. 2 we used the same procedure but did not include the highest-dose treatment. Imidacloprid has been found to occur in the nectar and pollen of crops from 1–50 ppb^59^, and at a maximum at 912 ppb^60^; see also Table S2 in Siviter *et al*. (2018) for average and maximum estimates of neonicotinoid concentrations in nectar ingested for a given honeybee and bumblebee foraging bout. We thus included the highest dose treatment in Exp. 1, which is still well below the LD50 dose for bumblebees^61^. All doses are within the range of those used in previous studies of acute imidacloprid effects on bee behaviour^34,35,37,62,63^.

All foragers we caught in containers consumed the solution (~10 seconds). We then blocked the opening of the container and kept bees in the dark for 1 hour prior to training. We did this to minimize activity and maximise pesticide absorption, the timing of which was in line with previous behavioural studies^37,63,64^. Post hoc analysis confirmed that the interval between dosing and the first floral visit was the same across treatments (see Table S2 for details on this and other data confirming timing elements did not differ between treatments).

### Experiment 1 Training and Testing

#### Training

Each bee was given a single training trial, during which it was presented with the 48-flower array, where one flower colour was rewarding and the other unrewarding. At the start of training, we removed the dark container, and gently transferred the bee to a single white pre-training flower, containing 4 µl of 50% (w/w) sucrose solution, stimulating its antennae with sucrose to induce proboscis extension. The vast majority of bees drank from this white flower, including all bees that went on to forage. A bee was allowed to visit as many flowers as it was motivated to visit, until it left the array, taking ~7-10 minutes. We re-filled flowers that bees visited after bees had visited 3–6 subsequent flowers. We re-filled in this manner to discourage bees from re-visiting flowers immediately, in order to encourage bees to learn colour rather than spatial cues. To prevent bees from learning the cue of the pipette as we re-filled flowers, we placed the pipette in the same number of unrewarding flower wells as well as rewarding ones each time we re-filled flowers (without pipetting any sucrose into unrewarding flowers). After a bee had finished its training trial, it either returned to the colony via the connecting tube, or we transferred it back to the colony if it left the array for >5 mins.

#### Testing

20 minutes after training, we tested bees on arrays that were identical to training arrays except that all flowers were unrewarding (containing 4 µl of water). The location of flower colours was randomly moved between each bee and between training and test trials, and flowers were wiped down with 70% ethanol in water between all trials. If bees did not return by themselves to the connecting tube to forage, then we did not test them.

We collected data on 20 bees per experimental treatment and 37 control bees, represented across 3 colonies and counter-balanced for the identity of the rewarding flower colour (Table 1). For the control treatment, we paired 12–13 bees with each experimental treatment. While our feeding and training regime likely resulted in minimal amounts of pesticide being brought back to the colony by subjects (the majority of the 20 µl should have been absorbed during the 1-hour period in the dark pre-training chamber^65^), we carried out these paired controls in case the neonicotinoid brought back affected other bees in the colony. This meant that on a given day, we only tested one experimental treatment group and paired controls, alternating the order we tested treatments across the three colonies. If any pesticide brought back to the colony affected the other foragers, we expected that the performance of control bees paired with each treatment group would differ; after confirming that this was not the case (Supplementary Material), we pooled the control bees for comparison to the experimental treatments.

**Table 1:** The sample size of bees that completed training in Experiment 1, across treatments (rows), colonies (columns) and colours trained to (numbers shown in smaller boxes to right of main number; above = bees trained that blue flowers were rewarding; below = bees trained that purple flowers were rewarding).

		Colony						
		1		2		3		Total
Treatment	Control	12	7	13	6	12	6	37
			5		7		6	
	0.22 ng	7	3	7	4	6	3	20
			4		3		3	
	0.45 ng	7	3	7	4	6	3	20
			4		3		3	
	1.12 ng	7	3	7	3	6	3	20
			4		4		3	

**Figure 3:**
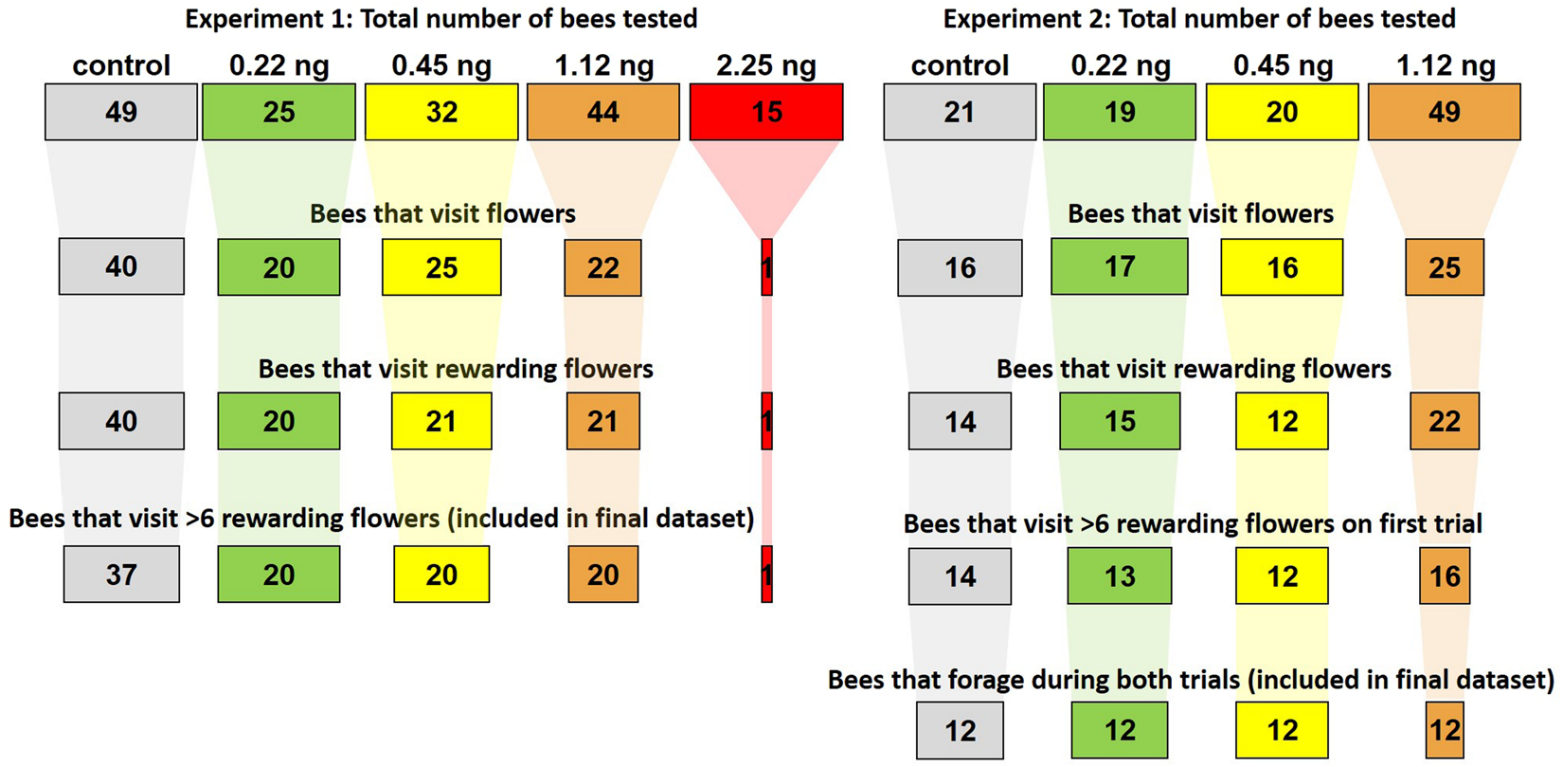
Summary of bee behaviour across both experiments. The numbers in cells are the sample size, while the width of the cell is the proportion of the original sample size. The data in the bottom rows was included in all foraging behaviour and learning analyses, with the exception of the 2.25 ng treatment in Experiment 1 (sample size too small).

We excluded the 2.25 ng treatment from Expt. 1 since the sample size of foraging bees was too small (see Fig. 3).

We excluded bees that either did not visit flowers within 15 minutes of being released into the arena, that only visited unrewarding flowers, or visited fewer than six rewarding flowers (for sample sizes by treatment, see Fig. 3).

### Experiment 2 Training and Testing

Our second experiment sought to replicate and expand Expt. 1, while giving bees a greater opportunity and motivation to learn. The four changes to our protocol were: (1) instead of using water as the CS-, we used 3% (w/w) NaCl solution; (2) bees experienced two training trials prior to the test trial; (3) if bees did not return to forage after completing their first trial, we returned them when possible, by allowing them to walk onto a spoonula in the colony and then transported them to the foraging arena (this was possible because these bees typically had very low activity levels) (Control n = 2, 0.45 ng n = 1; 1.12 ng n = 7). In these cases, the majority of the time (8/10) the bee initiated foraging once it was in the arena. Lastly, 4) we carried out a single control group rather than multiple controls as in Experiment 1, since control groups did not differ to each other in that experiment (Supplementary Material). Instead, all experimental and control treatments were inter-mixed across all days for each colony being tested.

We tested a total of 12 bees per treatment, represented across three colonies and counter-balanced for each colour being rewarding (Table 2). We did not test any bees in the 2.25 ng treatment, since Experiment 1 showed that the majority of the time these bees would not forage. As in that experiment, we excluded any bees that did not visit flowers within 15 minutes, did not gain rewards from more than six flowers, or that completed trial 1 but then did not return or would not forage in trial 2 (for sample sizes by treatment see Fig. 3).

**Table 2:** The sample size of bees that completed training in Experiment 2, across treatments (rows), colonies (columns) and colours trained to (numbers shown in smaller boxes to right of main number; above = bees trained that blue flowers were rewarding; below = bees trained that purple flowers were rewarding).

		Colony						
		1		2		3		Total
Treatment	Control	4	2	4	2	4	2	12
			2		2		2	
	0.22 ng	4	2	4	2	4	2	12
			2		2		2	
	0.45 ng	4	2	4	2	4	2	12
			2		2		2	
	1.12 ng	4	2	4	2	4	2	12
			2		2		2	

### Measurement and analysis of behavioural data

We filmed training and testing trials using an HD Sony camcorder (30 fps) mounted on a tripod placed on top of the arena pointing downwards. From the videos we used the behaviour coding program Solomon Coder (https://solomoncoder.com/), to code the time and type of flower visit each bee made to rewarding or unrewarding flowers. For the training trials, we coded whether 1) the flower the bee visited (landed on) was blue or purple; 2) it was rewarding (the bee consumed the sucrose); unrewarding (the bee probed the water with its proboscis); empty (the bee probed the sucrose well of a rewarding flower from which it had already consumed the sucrose); or didn’t drink (the bee landed on the flower but did not probe the sucrose well). We excluded ‘didn’t drink’ visits from the analysis of learning performance, since it could not be determined if this was a flower choice made in an attempt to gain nectar, or a random landing. We also excluded ‘empty’ visits since these were not reinforced. In the unrewarding test trial, we discriminated between types of visit further, defining three types of visit to a flower: ‘visiting the sucrose well’ was defined as the bee landing on a flower, and passing over the sucrose well, but not probing it with its proboscis (antennae/ legs could make contact), ‘probing’ a flower was defined as a bee landing on a flower, and probing the sucrose well with its proboscis (this was narrated on the video from live behavioural observation), and ‘didn’t drink’ was defined as the bee landing on the flower, but running over it without making any contact with the flower’s sucrose well. In the analysis of test performance we used the ‘visit sucrose well’ and ‘probe’ behaviours (pooled), because this seemed the most reliable measure of a bee’s choice, since bees can taste through their antennae and tarsi. We again excluded ‘didn’t drink’ visits from the analysis, since it could not be determined if this was a flower choice made in an attempt to gain nectar. We also carried out a more conservative analysis, using only the ‘probe’ data (i.e. the clearest measure of a bee’s choice), and found the same results (Supplementary Material).

To understand how multiple aspects of foraging behaviour might be altered by acute neonicotinoid exposure, we addressed the behaviours summarized in Fig. 2. For all data analyses, the two experiments were always considered separately rather than being pooled. All analyses were carried out in R v.3.4.3^66^. To carry out GLMMs we used the glmer() function in the lme4 package^67^ and to carry out LMMs we used the lme() function in the nlme package^68^. In cases where our models did not generate *p-*values (i.e. for GLMMs) we compared the fit of models using the anova() function to carry out a likelihood ratio test (LR test) between models with and without the variable in question. In all cases we ran full models initially, before removing non-significant interaction terms, while always including experimental variables. To carry out post-hoc tests on GLMMs to determine where significance lay between treatments, we used the package emmeans()^69^. Details on analyses for each question are given in the Supplementary Material.

## Results

### Tendency to initiate foraging (1)

**Figure 4:**
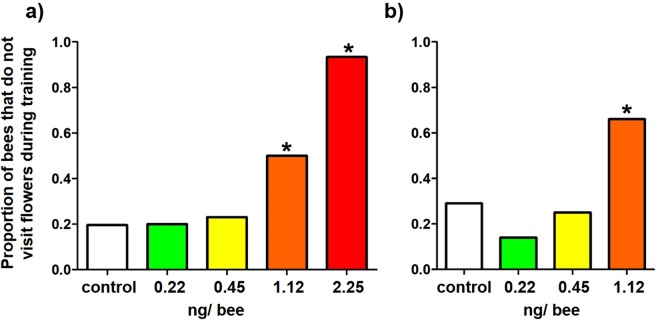
The proportion of bees in (**a**) Experiment 1 and (**b**) Experiment 2 that did not visit flowers during training, for each of the three experimental treatment groups. Asterisks above the bars indicate a significant difference compared to the control treatment.

Across both Experiment 1 and 2, bees in the higher-dose treatments were less likely to land on flowers at the start of the training trial. In Experiment 1, bees fed 2.25 ng or 1.12 ng were less likely to visit flowers compared to control bees (${{\rm{\chi }}}_{4}^{2}$ = 18.08; *p* < 0.005; 1.12 ng vs. control: ${{\rm{\chi }}}_{1}^{2}$ = 8.97; *p* < 0.005; 2.25 ng vs. control: ${{\rm{\chi }}}_{1}^{2}$ = 26.21; *p* < 0.001 (Bonferroni-adjusted α = 0.025) (Fig. 4a)), and in Experiment 2, bees fed 1.12 ng were less likely to visit flowers compared to control bees (${{\rm{\chi }}}_{3}^{2}$ = 15.55; *p* < 0.005; 1.12 ng vs. control: ${{\rm{\chi }}}_{1}^{2}$ = 4.70; *p* = 0.03) (Fig. 4b).

Of the bees that did visit flowers, treatment did not affect latency to their first floral visit (Expt. 1: *F*_*3*, *91*_ = 0.45, *p* = 0.72; Expt. 2: *F*_*3*, *41*_ = 0.32, *p* = 0.81).

### Success at collecting sucrose rewards (2)

**Figure 5:**
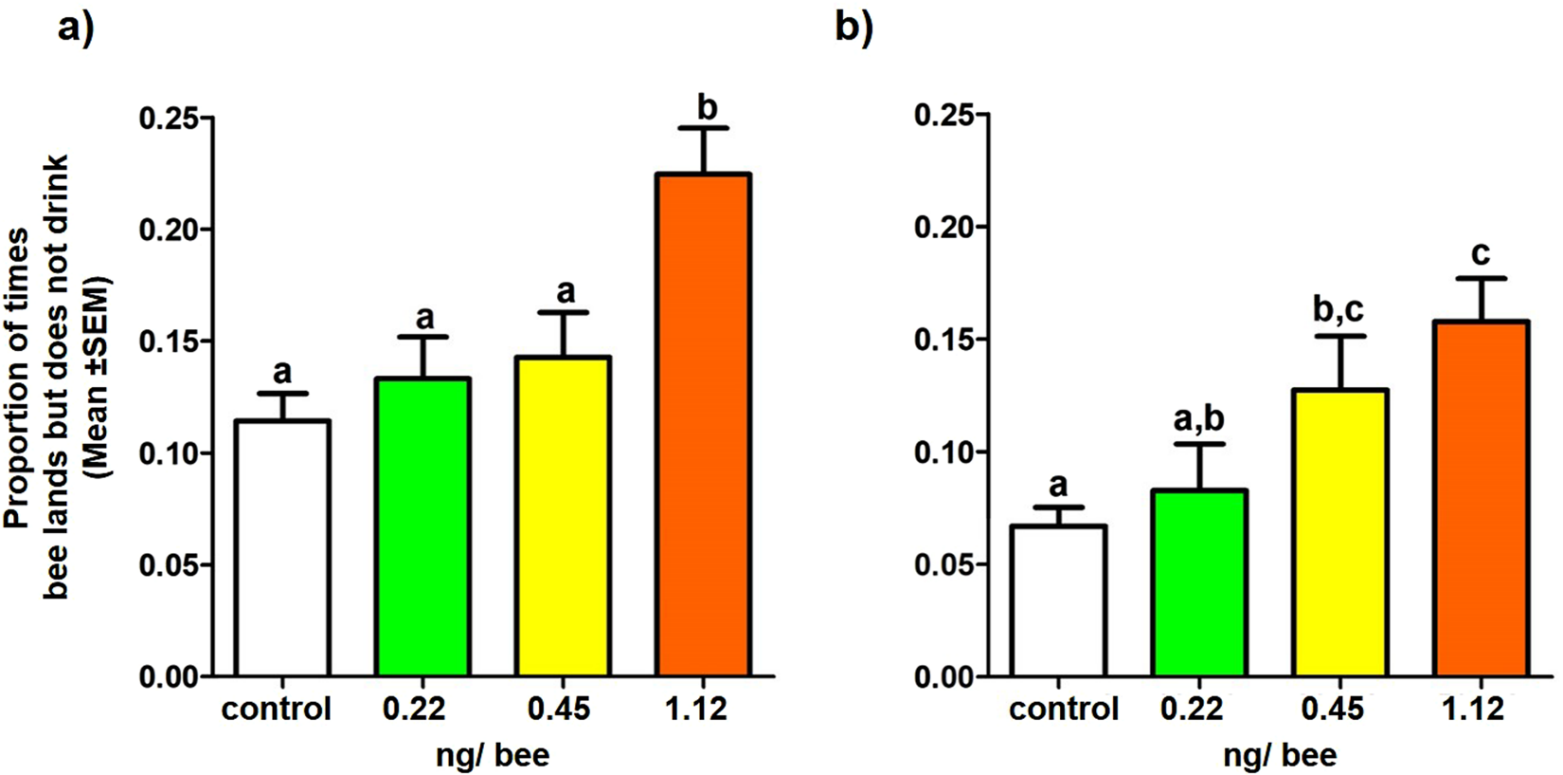
The mean ± SEM proportion of times a bee landed but did not drink from flowers (over the total number of visits it made) in (**a**) Experiment 1, (**b**) Experiment 2. For Experiment 1, this data included the single learning trial, while for Experiment 2 this included the two learning trials (data pooled here because the trials did not differ to each other). Letters indicate significant difference between treatment groups, within each experiment.

Overall, bees were more likely to not attempt to drink after landing on a flower in the pesticide-dosed treatments (Expt. 1: ${{\rm{\chi }}}_{3}^{2}$ = 21.21, *p* < 0.0001; Expt. 2: ${{\rm{\chi }}}_{3}^{2}$ = 19.85, *p* < 0.0005; post-hoc test results in Table S4; Fig. 5).

### Tendency to re-visit previously emptied flowers (3)

Pesticide exposure did not affect bees’ tendency to re-visit previously emptied flowers (Expt. 1: ${{\rm{\chi }}}_{3}^{2}$ = 3.36 *p* = 0.34; Expt. 2: ${{\rm{\chi }}}_{3}^{2}$ = 5.37, *p* = 0.15).

### Amount of sucrose collected (4)

**Figure 6:**
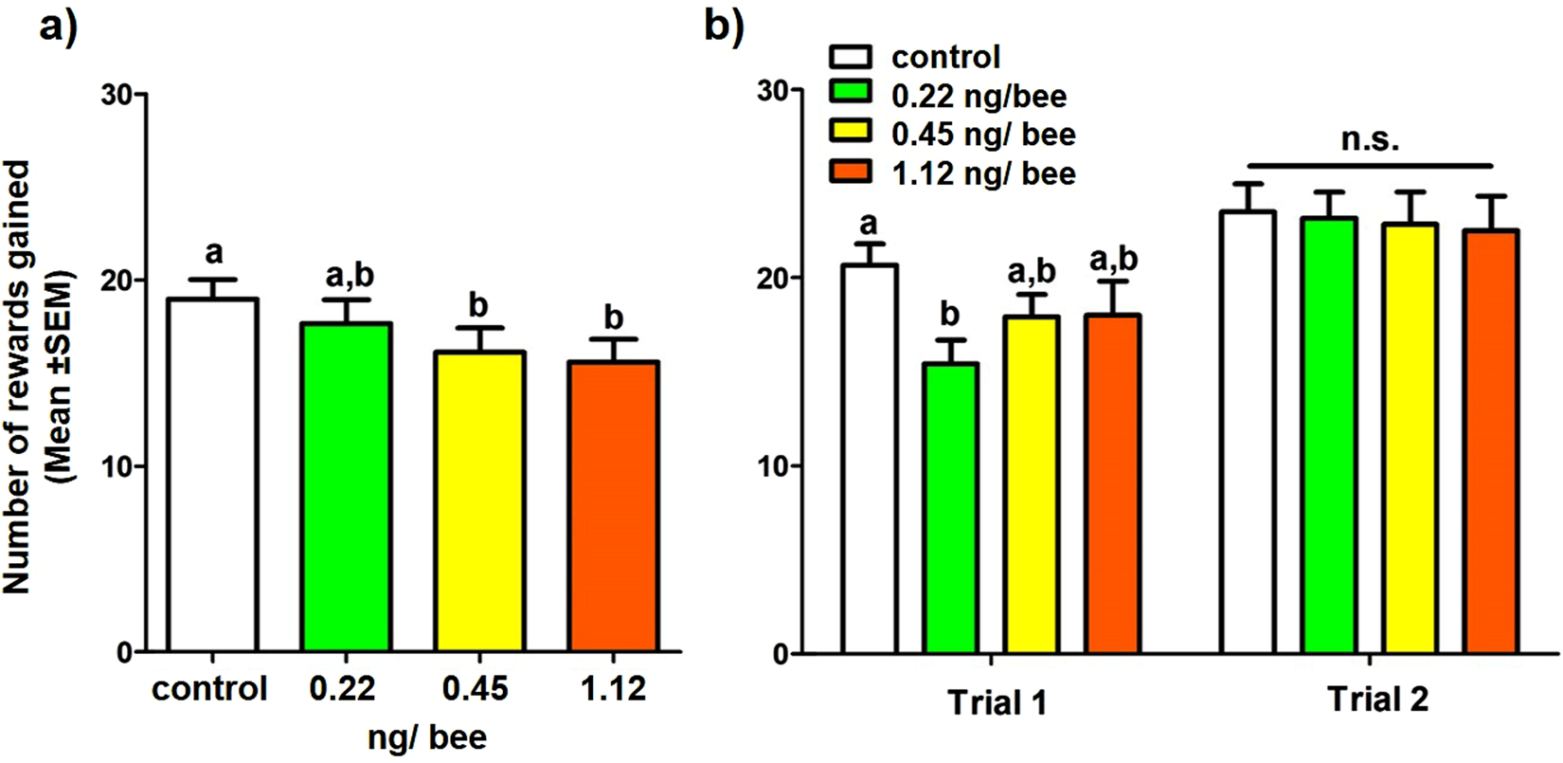
The number of flowers bees collected sucrose from (mean ± SEM), across the control and experimental treatments for (**a**) Experiment 1 and (**b**) Experiment 2. Letters indicate significant difference between treatment groups, within each experiment.

Amongst the bees that foraged sufficiently to be included in the learning analyses, in both Experiment 1 and 2, dosed bees made fewer visits where they collected sucrose, with the general relationship that the higher the pesticide dose, the fewer wells they collected sucrose from. In Experiment 1, bees in the 0.45 ng and 1.12 ng treatment group gained fewer rewards over the course of their foraging bout (${{\rm{\chi }}}_{3}^{2}$ = 11.61, *p* < 0.01; for post-hoc test results see Table S3a; Fig. 6a). In Experiment 2, bees in the 0.22 ng treatment gained fewer rewards than control bees in trial 1, but this effect had disappeared in trial 2 (for post-hoc results of trial × treatment interaction see Table S3b; Fig. 6b).

### Time spent foraging (5)

Across both experiments, bees did not differ between treatments in the amount of time they spent foraging. In Experiment 2, bees spent less time foraging in the second trial than the first (Expt. 1: treatment: *F*_*3*, *91*_ = 0.86, *p* = 0.47; Expt. 2: treatment: *F*_*3*, *41*_ = 0.96, *p* = 0.42; trial: *F*_*1*, *47*_ = 14.86, *p* < 0.0005).

### Learning performance (6)

Of the bees that foraged, treatment with neonicotinoids did not affect learning performance: while all bees showed evidence of learning (i.e. they improved from their first 10 landings to their last 10 landings), this did not differ between treatments, either in Experiment 1, or Experiment 2 (“visit block”: Expt. 1: ${{\rm{\chi }}}_{1}^{2}$ = 4.57, *p* = 0.033; Expt. 2: ${{\rm{\chi }}}_{1}^{2}$ = 4.84, *p* = 0.028; “treatment”: Expt. 1: ${{\rm{\chi }}}_{3}^{2}$ = 5.71, *p* = 0.13; Expt. 2: ${{\rm{\chi }}}_{3}^{2}$ = 0.78; *p* = 0.85; Fig. S2).

**Figure 7:**
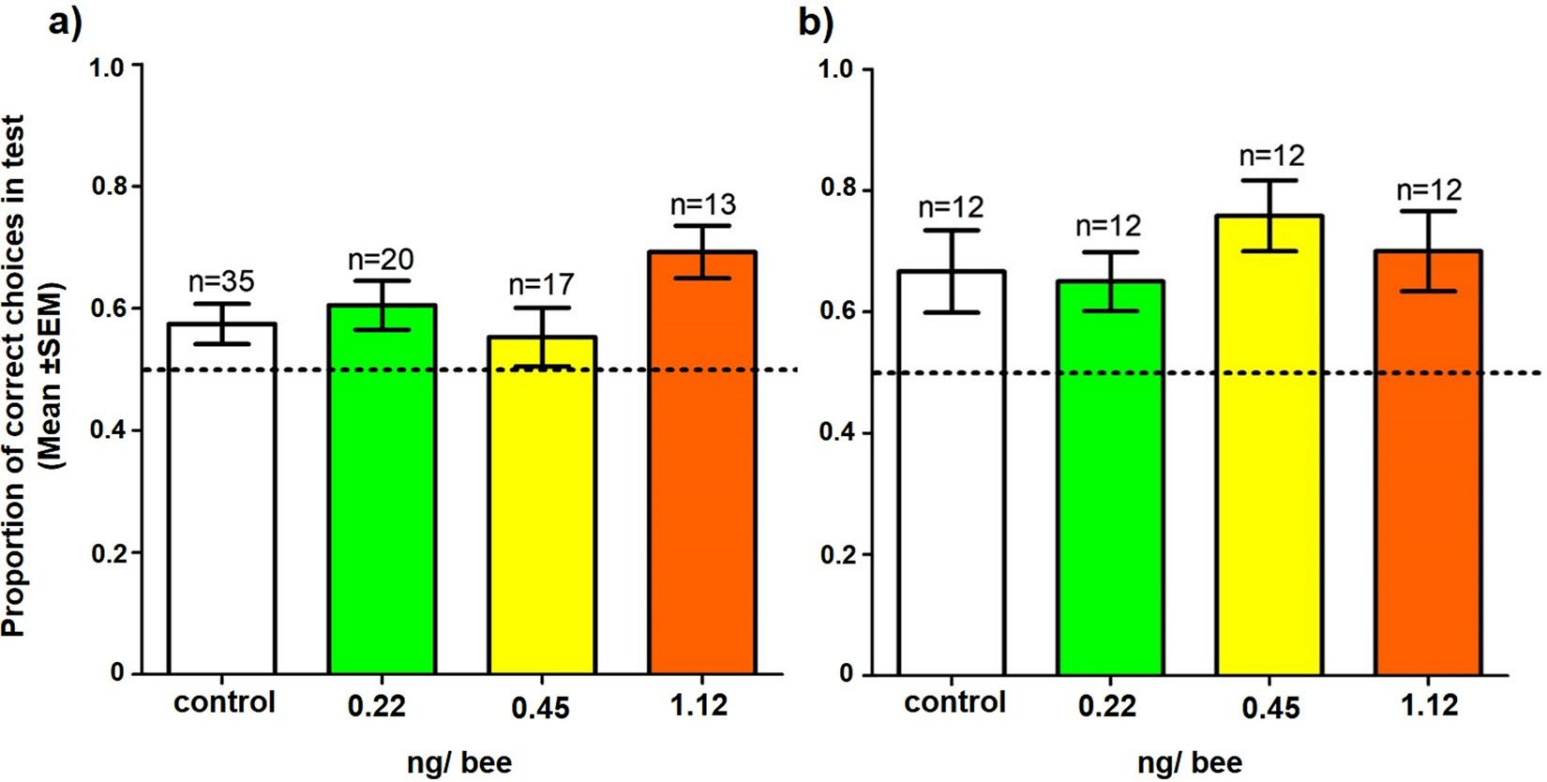
The proportion of correct choices (mean ± SEM) of bees in (**a**) Experiment 1 and (**b**) Experiment 2 of the first 10 flowers they visited in the test phase. Dotted line indicates chance performance. Treatments did not significantly differ to each other.

Similarly, test performance did not vary with pesticide treatment in either Experiment 1 or Experiment 2 (Expt. 1: ${{\rm{\chi }}}_{3}^{2}$ = 6.04, *p* = 0.11; Expt. 2: ${{\rm{\chi }}}_{3}^{2}$ = 2.22, *p* = 0.53; Fig. 7). This result held even if we only included visits to flowers where bees probed the flower (Supplementary Material, Fig. S3).

### Tendency to return to forage (7)

Of the bees that did visit flowers and complete a training trial in Experiment 1, bees in the 1.12 ng treatment were less likely to return for the test phase compared to control bees (${{\rm{\chi }}}_{1}^{2}$ = 6.47; *p* = 0.011). In these cases, bees returned to the colony but instead of carrying out “excited runs”^70^ to recruit foragers (typical of foraging bees returning^71^), they would instead often crawl underneath the colony and remain motionless. In Experiment 2, bees in the 1.12 ng treatment trended in the direction of being less likely to return to forage for a second trial by themselves: 2 control vs. 10 1.12 ng bees did not return by themselves (Fisher’s exact test: *p* = 0.078).

## Discussion

With both managed and wild bees facing many stressors^6^, it is important to fully understand the mechanisms driving performance impairments at the individual and colony levels. Unpacking the “black box” of sub-lethal effects on behaviour is essential for designing appropriate screening assays, and for implementing appropriate mitigation strategies^52^. Indeed, given regulatory changes in the use of neonicotinoid pesticides in some parts of the world, now is a critical time to be asking whether current approaches are optimal for estimating the magnitude of possible sub-lethal effects from alternative or newly developed pesticides bees may soon face^72^. Studies addressing the chronic, field-realistic exposure to any pesticide are a gold standard for estimating overall effects on colony foraging performance and success^15,16^. However, if we are to understand exactly how a pesticide affects bee behaviour and cognition, we need detailed behavioural experiments in controlled settings, paired with studies addressing effects at the neural level^25^. We asked if and how a neonicotinoid pesticide affects free-flying bees’ ability to learn about floral features, which allowed us to consider how acute exposure might impact not only the ability to learn, but also other foraging-related behaviour (Fig. 2). As a whole, our experiments demonstrated that a single acute dose of imidacloprid disrupted many foraging-related behaviours, but did not impair bees’ ability to learn an association between floral colour and a sucrose reward.

After ingesting a single dose of imidacloprid, one of the primary effects on behaviour was a reduction in bees’ apparent motivation to forage: in the higher-dose treatments, bees were less likely to visit flowers, collected less sucrose and were less likely to return to forage once having returned to the colony. Other studies have previously found that neonicotinoids suppress feeding motivation in bumblebees^48,49,73–75^. Free-flying studies have also found that neonicotinoids might cause a decrease in bumblebees’ tendency to forage for nectar (^76^ but see^14^); other studies have found that neonicotinoids reduce pollen foraging (^14–17,77^ but see^78^). Similarly, honeybees exposed to acute levels of imidacloprid were less likely to initiate foraging and made fewer foraging trips (^79^, see also^80–82^).

Another clear behavioural effect of imidacloprid was that, after landing on a flower, dosed bees were more likely to run over it without stopping to visit the sucrose well. This particular impairment persisted through both trials in Experiment 2 (some 90 minutes following pesticide exposure). Bypassing the nectar in this way may have been due to hyperactivity or impairment to motor coordination^83,84^. In line with previous findings in honeybees^83,85^, it seemed that in our study more broadly that bumblebees were initially hyperactive, followed by hypoactive. Indeed, bees in higher-dose treatments that did not land were often seen to fly or run around flowers for 5–10 minutes, followed by crawling into a corner of the arena and remaining motionless. Once back in the colony, individuals appeared to be less active: instead of moving around the colony in “excited runs”^70^ as newly returning foragers often do^71^, they would instead be stationary, or crawl underneath the colony. Interestingly, in Experiment 2, when we manually returned these foragers to the foraging arena for their second learning trial or test, these bees would generally commence foraging. Effects on activity levels or motor coordination could be detrimental both to bees’ ability to fly^85^ as well as to their ability to handle natural, morphologically complex flowers. Indeed, bumblebees exposed to chronic levels of thiamethoxam visited individual wildflowers flowers more times before gaining a nectar reward from them^78^, which could be evidence of motor deficits.

Despite the clear dose-dependent negative effects we found on a number of foraging-related behaviours, we did not find impairment to bees’ ability to learn which flowers were rewarding based on colour. This implies that at least in this scenario, foraging deficits are more likely to be due to the motivational and motor impairments we found, rather than the ability to learn. While a few studies have addressed acute effects of neonicotinoids on honeybee learning^35–37^, and a few have addressed the effects of chronic exposure to bumblebees^40,41,76,86^, we only found one study that addressed acute effects to bumblebee learning^40^. In that experiment, individual harnessed bumblebees were fed 10 μl of control, 2.4, 10 or 250 ppb thiamethoxam before being tested on a learning task an hour later. Bees in the two highest dose treatment groups had a lower “learning level” (the total number of times they extended their proboscis in response to the conditioned odour stimulus), and bees in the highest treatment group were less “trainable” (whether they learned the association or not over the 15 trials). There are a number of factors that differ between that study and our own, but we believe that the two most probable explanations of the difference in results are the conditioned stimuli the bees were trained to (olfactory versus visual) and the learning protocol (harnessed versus free-moving). These two features dominate studies of neonicotinoid effects on learning more broadly (Fig. 1; Table S1), and so they might also explain the difference in results in our study and the more general trend of results that find that neonicotinoids impair learning^18^.

With regard to differences in the modality of the conditioned stimulus, at least in honeybees, olfactory coding is impaired by a neonicotinoid^25^. This raises the possibility that bees may be less responsive to an olfactory stimulus because of difficulty in olfactory discrimination; if so, learning in other modalities may not be equivalently impaired. In order to determine this, one would need to carry out the same protocol but with conditioned stimuli in multiple modalities (e.g. visual vs. olfactory).

With regard to the second main difference in learning protocol, in our study bees were free-flying, as opposed to being immobilized in harnesses for PER. One limitation of the PER protocol is that it can often be difficult to differentiate between impairment to feeding/foraging motivation and learning performance, since both result in the same behavioural output: no proboscis extension to the trained stimulus. Researchers often attempt to control for this ambiguity by separately accounting for the effects of pesticide exposure on sucrose responsiveness; some have found that neonicotinoids reduce sucrose responsiveness^35,38,62^, while others have found no effect^37,40,41^. Even by excluding individuals that are less responsive to sucrose (as defined by some response threshold), there may still be motivational differences between treatment groups. For example, in our study, most of the bees we tested drank on the first (white) artificial flowers when we stimulated their antennae with sucrose, even though many of these bees did not then subsequently visit flowers. If this experiment had been in a PER setup, it is probable that many of these bees would have been included, since they would have been responsive to sucrose, but may have not been motivated enough to respond to an odour alone. In contrast to PER, in free-flying studies, behaviours associated with foraging motivation (i.e. propensity to visit flowers, amount of nectar collected) are different to the behaviour measured for learning performance (correct vs. incorrect floral choices), and thus motivation vs. learning performance can be more straightforwardly differentiated.

While the vast majority of studies investigating neonicotinoid effects on learning in bees have been in relation to olfactory stimuli (Fig. 1), there are three studies addressing effects on free-flying bees visiting visual stimuli^76,79^, which all have results compatible with our own, and bolster our finding that imidacloprid affects motivation more so than visual learning. In a recent study, *B*. *terrestris* chronically exposed to 1 ppb of imidacloprid were impaired in measures indicative of foraging motivation (time taken for bees to visit flowers and the number of flowers visited) but they were not impaired in their ability to learn which colour of flowers were rewarding^86^. In another recent study with *B*. *impatiens*, colonies that were chronically exposed to imidacloprid at 2.6 and 10 ppb were impaired in the number of trials that it took for individuals to develop preferences for the most highly rewarding flower (yellow) over three other less rewarding options^76^. While the reason behind this finding is not clear, the authors suggest it may be that treated bees were less motivated to forage (supported by the finding that they spent less time foraging and visited fewer flowers), and thus had less opportunity to learn. Finally, in a study with honeybees *A*. *mellifera*, individuals exposed to acute levels of imidacloprid were not impaired in their ability to learn that blue flowers were more highly rewarding than white, or in their ability to reverse this learned association^79^. However, interpretation of these results is difficult given that preference for blue flowers was affected by pesticide exposure when both flowers were equally rewarding.

Our study is a snapshot of the behavioural effects of a single dose of a neonicotinoid on an individual bee’s behaviour in the context of learning about colour, and of course many other factors could affect whether a neonicotinoid affects learning, such as the amount of time that a bee is exposed to a pesticide, the duration of time after exposure that we measure behaviour, as well as which neonicotinoid is used^87^. Even within a given pesticide treatment regime, all the factors known to affect learning, such as the salience of conditioned stimuli and motivational value of rewards^88^, might also affect our ability to detect pesticide effects on learning ability. In our experiment we chose stimuli that would be relatively difficult for bees to discriminate, to both be ecologically realistic (in natural foraging scenarios bees often need to discriminate between stimuli this similar in colour e.g.^89^), and to maximize the chances that we would detect an effect on learning if it existed in this context. We also replicated the experiment across two different scenarios where the motivational value of the US changed, as well as the opportunity to learn. These are factors that need to be considered in future studies if we are to determine not only if learning is impaired by a pesticide, but also whether such impairment has relevance for natural foraging scenarios, and thus what role such impairment might play relative to other effects on bee behaviour.

More broadly, our study highlights some of the factors that need to be considered when addressing the effects of a stressor on cognition. Specifically, we need to address cognition across more than a single (usually olfactory) modality, including consideration of how more realistic multi-modal floral stimuli^90^ might help compensate for foraging performance deficits previously documented in single-modality scenarios. We also need to address a broader range of cognitive abilities and learning scenarios. Given that bees use a wide range of cognitive abilities when foraging, from spatial cognition^64^ to learning non-elemental (configural) associations^27,91^, by focusing just on olfactory associative learning we are likely only seeing a part of the picture. In addition, a single protocol can only ever tell us about animal behaviour under certain conditions, and so a broader range of behavioural protocols, especially ones that address cognition under more ecologically realistic scenarios will be informative.

## Supplementary information

supplementary material

## Data Availability

Data will be made available in Dryad.
