# Supplementary material for: A neonicotinoid pesticide impairs foraging, but not learning, in free-flying bumblebees
Source: Sci Rep. 2019 Mar 18;9:4764. doi: 10.1038/s41598-019-39701-5 (PMC6423345; doi:10.1038/s41598-019-39701-5)
Supplement: Supplementary file 1 — supplementary material [file 41598_2019_39701_MOESM1_ESM.docx]

**Supplementary Material for:**

**A neonicotinoid pesticide impairs foraging,**

**but not learning, in free-flying bumblebees**

Muth, F.*^1^, Leonard, A.S.^1^

^1^ Department of Biology, University of Nevada, Reno, NV, 89557.

*Corresponding author:

Felicity Muth,

Department of Biology,

1664 N Virginia Street,

University of Nevada,

Reno, NV 89557.

[fmuth@unr.edu](mailto:Felicitymuth@email.arizona.edu)

520-612-3801

**Table S1:** Summary of studies (n=50) addressing pesticide (and pesticide-related substance) effects on learning. Studies were identified based on a Web of Science and Google Scholar search of “pesticide bee learning” of all dates until June 2018.

| **Paper** | **Bee** | **pesticide type** | **learning protocol** | **conditioned stimulus** |
| --- | --- | --- | --- | --- |
| Abramson et al., 1999 | *Apis mellifera* | organochloride, pyrethroid, carbamate | PER | odour |
| Abramson et al., 2004 | *Apis mellifera* | insect growth regulator | PER | odour |
| Abramson et al., 2012 | *Apis mellifera* | pyridine azomethine | PER | odour |
| Aliouane et al., 2009 | *Apis mellifera* | neonicotinoid | PER | odour |
| Alkassab and Kirchner, 2016 | *Apis mellifera* | neonicotinoid | PER | odour |
| Bonnafé et al., 2017 | *Apis mellifera* | monoterpenoids | PER | odour |
| Chakrabarti et al., 2015 | *Apis cerana* | organophosphosphate, pyrethroid, organochloride | PER | odour |
| Ciarlo et al., 2012 | *Apis mellifera* | various spray adjuvants, neonicotinoid | PER | odour |
| Decourtye et al., 2003 | *Apis mellifera* | neonicotinoid | PER | odour |
| Decourtye et al., 2004a | *Apis mellifera* | neonicotinoid | PER | odour |
| Decourtye et al., 2004b | *Apis mellifera* | neonicotinoid, pyrethroid | PER and free-flying | odour |
| Decourtye et al., 2005 | *Apis mellifera* | 9 pesticides | PER | odour |
| El Hassani et al., 2008 | *Apis mellifera* | neonicotinoid | PER | odour |
| Frost et al., 2013 | *Apis mellifera* | pyrethroid | PER | odour |
| Han et al., 2010 | *Apis mellifera* | neonicotinoid | PER and free-crawling | odour and colour |
| Herbert et al., 2014 | *Apis mellifera* | glyphosphate | PER | odour |
| Hesselbach and Scheiner, 2018 | *Apis mellifera* | butenolide | PER | odour |
| Jin et al., 2015 | *Osmia cornuta* | neonicotinoid | free-crawling | spatial location |
| Karahan et al., 2015 | *Apis mellifera* | neonicotinoid | free-flying | colour |
| Li et al., 2017 | *Apis mellifera and A. cerana* | organophosphate | PER | odour |
| Liao et al., 2018 | *Apis mellifera* | pyrethroid | PER | odour |
| Mamood and Waller, 1990 | *Apis mellifera* | pyrethroid | PER | odour |
| Mengoni Goñalons and Farina, 2015 | *Apis mellifera* | neonicotinoid | PER | odour |
| Mengoni Goñalons and Farina, 2018 | *Apis mellifera* | neonicotinoid, glyphosphate | PER | odour |
| Nakasu et al., 2014 | *Apis mellifera* | Protein-fusion biopesticide | PER | odour |
| Papach et al., 2017 | *Apis mellifera* | neonicotinoid | PER | odour |
| Pham-Delegue et al., 2000 | *Apis mellifera* | serine proteinase inhibitors | PER | odour |
| Phelps et al., 2018 | *Bombus impatiens* | neonicotinoid | free-flying | colour |
| Piiroinen et al., 2016 | *Bombus terrestris* | neonicotinoid | PER | odour |
| Piiroinen and Goulson, 2016 | *Bombus terrestris* and *Apis mellifera* | neonicotinoid | PER | odour |
| Ramirez-Romero et al., 2005 | *Apis mellifera* | neonicotinoid, pyrethroid | free-flying | odour |
| Ramirez-Romero et al., 2008 | *Apis mellifera* | neonicotinoid | PER | odour |
| Rix and Christopher Cutler, 2016 | *Apis mellifera* | formamidine | PER | odour |
| Samuelson et al., 2016 | *Bombus terrestris* | neonicotinoid | free-flying | spatial location |
| Schneider et al., 2012 | *Apis mellifera* | oxalic acid | PER | odour |
| Stanley et al., 2015 | *Bombus terrestris* | neonicotinoid | PER | odour |
| Stone et al., 1997 | *Apis mellifera* | organochloride | PER | odour |
| Tan et al., 2013 | *Apis cerana* | pyrethroid | PER | odour |
| Tan et al., 2015 | *Apis cerana* | neonicotinoid | PER | odour |
| Tan et al., 2017 | *Apis cerana* | butenolide | PER | odour |
| Taylor et al., 1987 | *Apis mellifera* | pyrethroid | PER | odour |
| Thany et al., 2015 | *Apis mellifera* | pyrethroid, neonicotinoid | PER | odour |
| Tison et al., 2017 | *Apis mellifera* | neonicotinoid | PER | odour |
| Urlacher et al., 2016 | *Apis mellifera* | organophosphate | PER | odour |
| Weick and Thorn, 2002 | *Apis mellifera* | organophosphate | PER | odour |
| Williamson and Wright, 2013 | *Apis mellifera* | neonicotinoid, organophosphate | PER | odour |
| Williamson et al., 2013 | *Apis mellifera* | neonicotinoid, organophosphate | PER | odour |
| Wright et al., 2015 | *Apis mellifera* | neonicotinoid | PER | odour |
| Yang et al., 2012 | *Apis mellifera* | neonicotinoid | PER | odour |
| Zhang and Nieh, 2015s | *Apis mellifera* | neonicotinoid | SER (sting extension reflex) | odour |

**Methods**

*Pre-training protocol*

Colonies were initially given access to a white wicked feeder offering 30% (w/w) sucrose, in order for bees to learn to visit a feeder in the arena and return to their colony. Once we had ~20 foragers visiting this feeder, we replaced it with a pre-training array. This array consisted of 14 flowers, made from plastic tubes (D x H: 3.3 x 7cm), with human-white caps. Each of these caps contained a central well offering 20µl 30% (w/w) sucrose which we re-filled as bees visited the flowers. As foragers visited these shaping flowers, we marked their thorax with paint of different colours to identify individuals. Once we had foragers visiting the pre-training array, we restricted the colony’s access to this array to <1 hour a day. We pipetted 30% (w/w) sucrose directly into honeypots (2-6) at the end of each experimental day such that the colony’s stores would be almost empty the next morning, and workers would be motivated to forage.

*Body size measures*

To ensure that bee body size did not differ between treatments we euthanized all bees after their trial by freezing, and then measured inter-tegular span and head width as proxies of body size (e.g. Hagen and Dupont, 2013).

*Artificial flower colours:* We chose human-blue and purple flowers for the colour learning discrimination in the current study: pilot data suggested that bees did not have a strong preference for one of these colours over the other, and that an association could be learned in a single foraging trial. We measured the reflectance spectra of the two colours and the irradiance in the experimental arena using a Jaz UV-VIS spectrophotometer and SpectraSuite software (Ocean Optics, Dunedin, FL, U.S.A.). We then plotted these spectra into bee colour space (Chittka, 1992) taking into account the photoreceptor spectral sensitivities of *B. impatiens* (Skorupski & Chittka, 2010) and lighting conditions, using AVICOL v.6 (AVICOL: A program to analyse spectrometric data; free program available from the author at [dodogomez@yahoo.fr](mailto:dodogomez@yahoo.fr)).


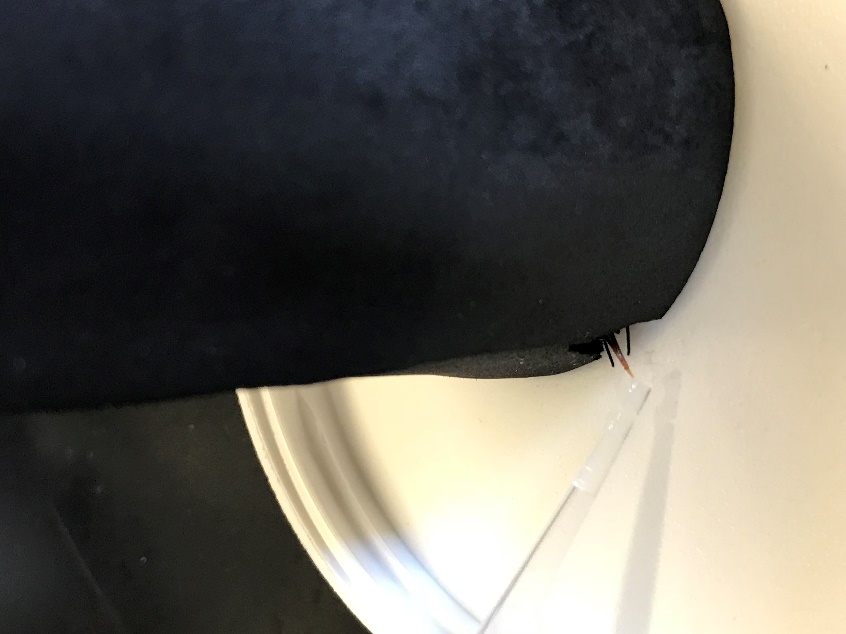

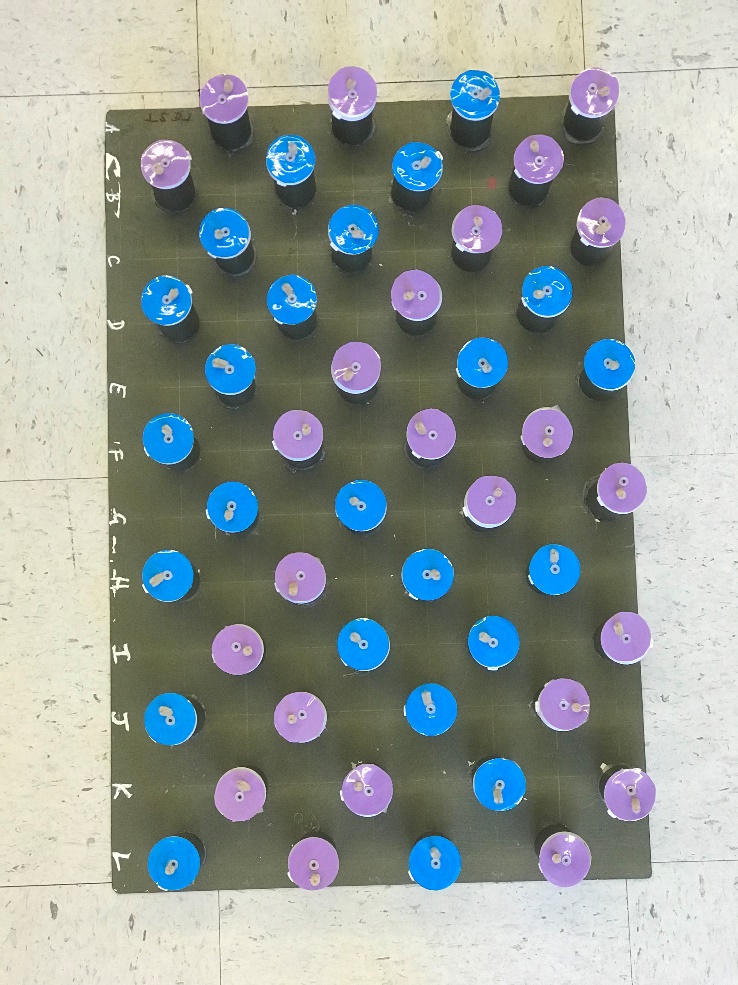


**b)**

**a)**

**proboscis**

**pipette**


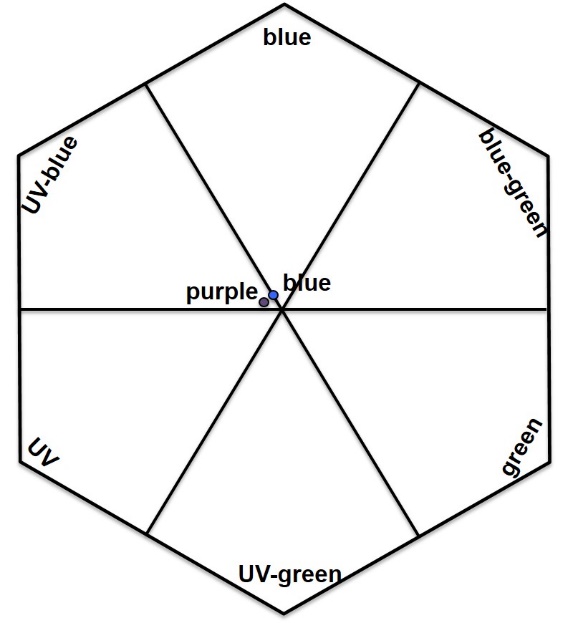

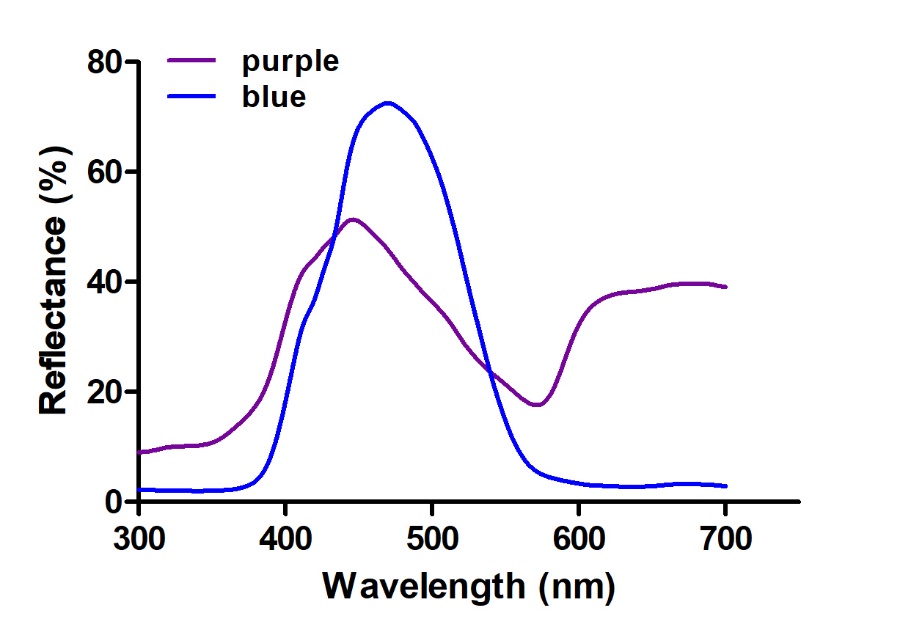


**d)**

**c)**

**Figure S1**: The experimental set-up: a) a top-down view of the foraging array; b) the holding chamber where bees were pre-fed with a neonicotinoid or control solution before being held for 1 hour (photo shows a bee being fed sucrose via a pipette). c) Reflectance spectra and d) bee colour space representation of the two colours of stimuli bees were trained to in the current experiment, chromatic contrast = 0.038.

*Data analysis*

Details for each question addressed:

*1) Tendency to initiate foraging:* To determine whether the propensity of bees to forage depended on the treatment group, we carried out Chi-square tests comparing the number of bees (in trial 1) that did not visit flowers to the number of bees that foraged (collecting more than 6 rewards, Fig. 3), comparing each experimental treatment to the control group.

To determine whether bees differed in their time they took to land on flowers between treatments, we carried out LMMs with the response variable “time to land” (in minutes) and the explanatory factors “treatment”, “colour trained to” and the random factor “colony”.

*2) Success at collecting sucrose rewards*: For this analysis and analyses described below, we only included bees that met the criteria to be included for learning analyses (i.e. as shown in Figure 3). After noticing that bees would frequently land on flowers, but then run over them without making contact with the sucrose well, we aimed to investigate whether the prevalence of this behaviour differed between treatments. We ran binomial GLMMs with the response variable “didn’t drink” (see “*Measurement and analysis of behavioural data*” section of manuscript for definitions) vs. all other types of visit (rewarded/ unrewarded/ empty) and the explanatory variables “treatment”, “colour rewarding”, and the random factors “bee” and “colony”. For Experiment 1, this data included the single learning trial, and for Experiment 2, we included “trial” (1 or 2) as an explanatory factor.

*3) Tendency to re-visit previously emptied flowers:* To determine whether treatments differed in the proportion of flowers they re-visited, we ran binomial GLMMs with the response variable “number of empty flowers probed” vs. all other types of visit (rewarded/ unrewarded/ didn’t drink), the explanatory factors “treatment” and “trial” (Expt. 2 only), and the random factors “colony” and “bee” (Expt. 2 only).

*4) Amount of sucrose collected*: To determine if the pesticide affected how many sucrose rewards bees gained from flowers across the foraging bout, we ran GLMMs with Poisson distributions with the response variable “number of flowers gained sucrose from” and the explanatory variables “treatment”, “colour rewarding”, “trial” (Expt. 2 only) and the random factors “colony” and “bee” (Expt. 2 only).

*5) Time spent foraging:* To determine if the pesticide affected how long the bee spent foraging, we carried out LMMs with the response variable “time spent foraging” (in minutes) and the explanatory factors “treatment”, “colour trained to” and the random factor “colony”. For Experiment 2, we also included the explanatory factor “trial” and random factor “bee”.

6) *Learning performance*: To determine whether bees differed in their ability to learn colour associations we carried out two analyses. First, we compared the number of correct choices bees made during their first 10 visits bees made to the last 10 visits; we did this because bees in the different treatments differed in how many visits they made (see *Amount of Sucrose collected* section of results). To do this, we ran a GLMM with a Poisson distribution with the response variable “number of correct choices” and the explanatory variables “treatment” (control, 0.22 ng, 0.45 ng or 1.12 ng), “visit block” (either the first 10 visits or the last 10 visits), “colour rewarding” (blue or purple) and the random factors “bee” and “colony”. For this analysis we excluded all “didn’t drink” visits to flowers (defined in the *Measurement and analysis of behavioural data* section), since it could not be determined if this was a flower choice made in an attempt to gain nectar or instead a random landing. We also excluded ‘empty’ visits, since these were not reinforced. Data from bees that made fewer than 20 visits to flowers were not included in the analysis.

We then compared bees’ performance in the test phase, addressing the first 10 visits to flowers a bee made (including both “visits to sucrose well” and “probes” as defined in the *Measurement and analysis of behavioural data* section, although results did not change if we only addressed “probes” (Fig. S3). We ran a GLMM with a binomial distribution where the response variable was correct/incorrect and the explanatory variables were “treatment”, “colour rewarding”, and the random factors “bee” and “colony”. Bees that did not return for the test phase, or visited fewer than 10 visits to flowers in the test phase were included in the “trial” learning analyses, but excluded from test performance analyses (Expt. 1: control n=2; 0.45 ng; n=3, 1.12 ng n=7).

*7) Tendency to return to forage:* To compare the number of bees that did not return to forage for the test or the second trial (Expt. 2), we used Chi-square and Fisher Exact tests.

**Results**

*Body size*

To limit body size effects, we selected foragers that appeared to be roughly the same size and indeed our final dataset of bees included in the experiment did not differ in size between treatments (LMM with the response variable “head width” or “inter-tegular distance” and the explanatory variable “treatment” and random factor “colony”: Expt. 1: No treatment effect on head size: *F_3, 90_* = 1.42, *p* = 0.24; No treatment effect on inter-tegular width: *F_3, 89_* = 1.96, *p* = 0.13; Expt. 2: No treatment effect on head size: *F_3, 41_* = 1.24, *p* = 0.31; No treatment effect on inter-tegular width: *F_3, 41_* = 1.54, *p* = 0.22).

We also tested whether bees that foraged were larger than bees that did not (i.e. not landing, not gaining any nectar, or gaining nectar from <6 flowers). We did this for the 1.12 ng treatment only, in Experiment 1, since sample sizes were too small in other treatments. While we found that there was a small trend in the direction of smaller foragers not foraging once having consumed 1.12 ng IMD, this relationship was not significant (No treatment effect on head size: *F_1, 40_* = 2.37, *p* = 0.13; No treatment effect on inter-tegular width: *F_1, 39_* = 2.81, *p* = 0.10).

*Timing*

Because timing is key for both pesticide effects and effects on learning, we ensured that timings did not differ between different parts of the experiment. Precise timings are given below in **Table S2** (means±SEM):

|  | **Experiment 1** | **Experiment 2** |
| --- | --- | --- |
| Time between feeding and training | control: 59.51±1.15;  0.22 ng: 59.15±1.17;  0.45 ng: 58.85±1.48;  control: 56.38±1.24;  2.25 ng: 60.00±1.73 | control: 62±2.05;  0.22 ng: 63±2.10;  0.45 ng: 60±1.80;  1.12 ng: 62±2.54 |
| Time between being released from the container and probing their first flower | control: 4.30±0.84;  0.22 ng: 4.00±0.65;  0.45 ng: 5.00±0.82;  1.12 ng: 4.15±0.78 |  |
| Time spent foraging on first trial | control: 10.00±1.63;  0.22 ng: 8.15±0.75;  0.45 ng: 7.60±0.70;  1.12 ng: 9.65±1.65 |  |
| Time between bee leaving the array on trial 1 and commencing foraging on trial 2 | N/A | control: 14±1.16;  0.22 ng: 12±0.71;  0.45 ng: 12±1.44;  1.12 ng: 15±1.36 |
| Time between bee leaving the final trial and probing the first flower in the test trial | control: 18.89±1.38;  0.22 ng: 20.55±0.92;  0.45 ng: 20.75±1.05;  1.12 ng: 19.80±1.45 | control: 18±2.11;  0.22 ng: 21±0.65;  0.45 ng: 22±1.01; 1.12 ng:21±0.47 |

*Experiment 1, Control treatments*

In Experiment 1, we carried out three control groups paired to three experimental treatment groups (0.22 ng, 0.45 ng and 1.12 ng IMD), to control for any possible effects of the treatment we assigned bees to on a given day affecting the other bees in the colony (through the bees returning to the colony after trial 1 with the pesticide they had been fed in their crop). However, we did not find that control groups varied in any of the metrics that experimental groups varied in, so these control groups were pooled for comparison to experimental treatments. Specifically, bees in the three control groups did not differ in their propensity to visit flowers (χ^2^ test_:_ χ^2^_2_ = 1.635, *p* = 0.442), in how many nectar rewards they gained (GLM: *F^­^_2, ­34_ =* 1.43, *p* = 0.311), in their tendency to run across flowers without probing (GLM: *F^­^_2, ­34_ =* 1.202, *p* = 0.313), or in the total number of flowers they visited (GLM: *F^­^_2, ­34_ =* 0.899, *p* = 0.416).

*Foraging behaviour*

**Table S3**: Post-hoc test of total number of nectar rewards gained:

1. Experiment 1:

| Treatment | emmean | SE | asymp.LCL | asymp.UCL | group |
| --- | --- | --- | --- | --- | --- |
| Control | 2.95 | 0.10 | 2.75 | 3.16 | 1 |
| 0.22 ng | 2.85 | 0.11 | 2.64 | 3.07 | 1,2 |
| 0.45 ng | 2.76 | 0.11 | 2.54 | 2.98 | 2 |
| 1.12 ng | 2.77 | 0.11 | 2.55 | 2.99 | 2 |

1. Experiment 2:

| Trial | Treatment | emmean | SE | asymp.LCL | asymp.UCL | group |
| --- | --- | --- | --- | --- | --- | --- |
| 1 | Control | 3.02 | 0.078 | 2.87 | 3.17 | 2 |
| 1 | 0.22 ng | 2.72 | 0.086 | 2.55 | 2.89 | 1 |
| 1 | 0.45 ng | 2.87 | 0.082 | 2.71 | 3.034 | 1,2 |
| 1 | 1.12 ng | 2.88 | 0.082 | 2.72 | 3.036 | 1,2 |

| Trial | Treatment | emmean | SE | asymp.LCL | asymp.UCL | group |
| --- | --- | --- | --- | --- | --- | --- |
| 2 | Control | 3.15 | 0.075 | 3.00 | 3.29 | 1 |
| 2 | 0.22 ng | 3.13 | 0.075 | 2.98 | 3.28 | 1 |
| 2 | 0.45 ng | 3.12 | 0.075 | 2.97 | 3.26 | 1 |
| 2 | 1.12 ng | 3.10 | 0.076 | 2.95 | 3.25 | 1 |

**Table S4**: Post hoc test of bees’ tendency to visit flowers without probing the sucrose well:

1. Experiment 1:

| Treatment | emmean | SE | asymp.LCL | asymp.UCL | group |
| --- | --- | --- | --- | --- | --- |
| Control | -2.10 | 0.14 | -2.38 | -1.81 | 1 |
| 0.22 ng | -1.93 | 0.17 | -2.26 | -1.60 | 1 |
| 0.45 ng | -1.79 | 0.17 | -2.13 | -1.45 | 1 |
| 1.12 ng | -1.29 | 01.6 | -1.61 | -0.97 | 2 |

1. Experiment 2:

| Trial | Treatment | emmean | SE | asymp.LCL | asymp.UCL | group |
| --- | --- | --- | --- | --- | --- | --- |
| 1 | Control | -2.79 | 0.30 | -3.37 | -2.20 | 1 |
| 1 | 0.22 ng | -2.65 | 0.31 | -3.25 | -2.05 | 1,2 |
| 1 | 0.45 ng | -2.10 | 0.29 | -2.67 | -1.53 | 2,3 |
| 1 | 1.12 ng | -1.89 | 0.28 | -2.45 | -1.34 | 3 |

| Trial | Treatment | emmean | SE | asymp.LCL | asymp.UCL | group |
| --- | --- | --- | --- | --- | --- | --- |
| 2 | Control | -2.707488 | 0.30 | -3.29 | -2.12 | 1 |
| 2 | 0.22 ng | -2.568524 | 0.30 | -3.16 | -1.98 | 1,2 |
| 2 | 0.45 ng | -2.020313 | 0.29 | -2.59 | -1.45 | 2,3 |
| 2 | 1.12 ng | -1.815433 | 0.29 | -2.37 | -1.26 | 3 |

*Learning performance*


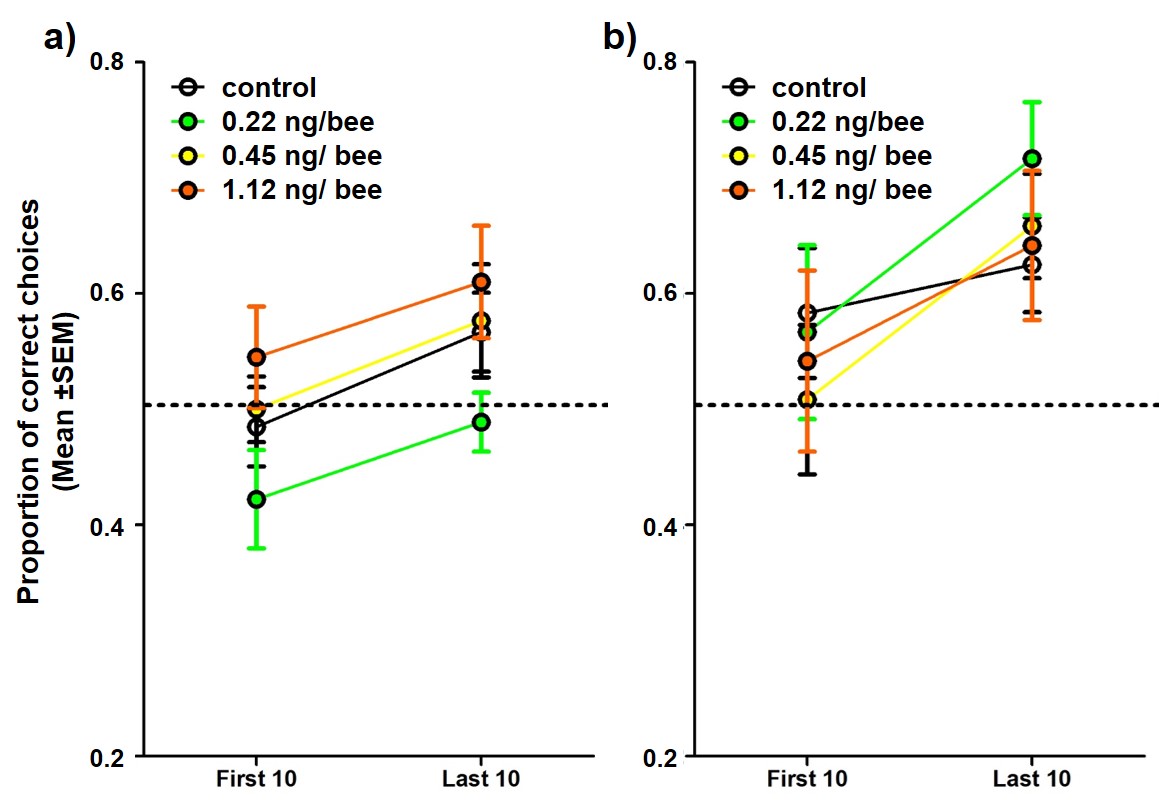


**Figure S2**: The proportion (mean ± SEM) of correct choices bees made in their first 10 visits to flowers and last 10 visits to flowers, in a) Experiment 1 and b) Experiment 2. Dotted line indicates chance performance. Treatments did not significantly differ to each other.

*Test phase*

In addition to addressing visits to flowers’ sucrose wells (main article) as a measure of choice, we also separately addressed a subset of visits, where bees probed the sucrose well on the flower, as a more conservative measure. We found that using this measure resulted in the same findings: bees did not differ in their test performance across the different treatment groups (comparison of models with and without “treatment”: Expt. 1: χ^2^_3_ = 5.24, *p* = 0.16; Expt. 2: χ^2^_3_ = 5.24, 3.19, *p* = 0.36; Figure S1).


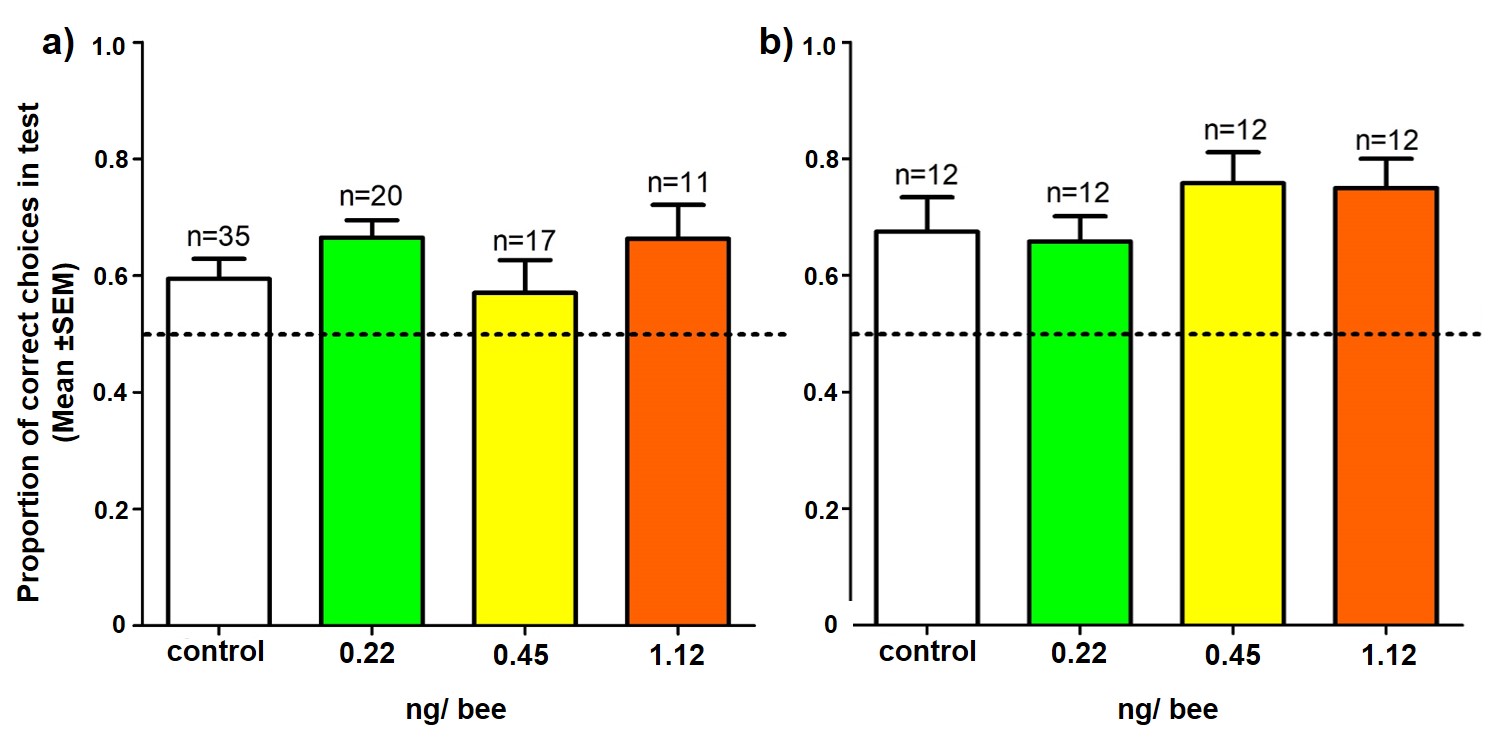


**Figure S3**: The proportion of correct choices (mean ± SEM) in a) Experiment 1 and b) Experiment 2 to flowers in the test phase, of the first 10 visits made. Here, “choice” was specifically coded as bees probing the sucrose well on flowers. Dotted line indicates chance performance. Treatments did not significantly differ to each other.

**References**

**Abramson, C. I., Aquino, I. S., Ramalho, F. S. and Price, J. M.** (1999). The Effect of Insecticides on Learning in the Africanized Honey Bee (*Apis mellifera L*.). *Arch. Environ. Contam. Toxicol.* **37**, 529–535.

**Abramson, C. I., Squire, J., Sheridan, A. and Mulder, P. G.** (2004). The Effect of Insecticides Considered Harmless to Honey Bees (*Apis mellifera*): Proboscis Conditioning Studies by Using the Insect Growth Regulators Tebufenozide and Diflubenzuron. *Environ. Entomol.* **33**, 378–388.

**Abramson, C. I., Sokolowski, M. B. C., Brown, E. A. and Pilard, S.** (2012). The effect of pymetrozine (Plenum WG-50®) on proboscis extension conditioning in honey bees (*Apis mellifera*: Hybrid var. Buckfast). *Ecotoxicol. Environ. Saf.* **78**, 287–295.

**Aliouane, Y., el Hassani, A. K., Gary, V., Armengaud, C., Lambin, M. and Gauthier, M.** (2009). Subchronic exposure of honeybees to sublethal doses of pesticides: Effects on behavior. *Environ. Toxicol. Chem.* **28**, 113.

**Alkassab, A. T. and Kirchner, W. H.** (2016). Impacts of chronic sublethal exposure to clothianidin on winter honeybees. *Ecotoxicology* **25**, 1000–1010.

**Bonnafé, E., Alayrangues, J., Hotier, L., Massou, I., Renom, A., Souesme, G., Marty, P., Allaoua, M., Treilhou, M. and Armengaud, C.** (2017). Monoterpenoid-based preparations in beehives affect learning, memory, and gene expression in the bee brain. *Environ. Toxicol. Chem.* **36**, 337–345.

**Chakrabarti, P., Rana, S., Bandopadhyay, S., Naik, D. G., Sarkar, S. and Basu, P.** (2015). Field populations of native Indian honey bees from pesticide intensive agricultural landscape show signs of impaired olfaction. *Sci. Rep.* **5**, 12504.

**Ciarlo, T. J., Mullin, C. A., Frazier, J. L. and Schmehl, D. R.** (2012). Learning Impairment in Honey Bees Caused by Agricultural Spray Adjuvants. *PLoS One* **7**, e40848.

**Decourtye, A., Lacassie, E. and Pham-Delègue, M.-H.** (2003). Learning performances of honeybees (*Apis mellifera L*) are differentially affected by imidacloprid according to the season. *Pest Manag. Sci.* **59**, 269–278.

**Decourtye, A., Armengaud, C., Renou, M., Devillers, J., Cluzeau, S., Gauthier, M. and Pham-Delègue, M.-H.** (2004a). Imidacloprid impairs memory and brain metabolism in the honeybee (*Apis mellifera L*.). *Pestic. Biochem. Physiol.* **78**, 83–92.

**Decourtye, A., Devillers, J., Cluzeau, S., Charreton, M. and Pham-Delègue, M.-H.** (2004b). Effects of imidacloprid and deltamethrin on associative learning in honeybees under semi-field and laboratory conditions. *Ecotoxicol. Environ. Saf.* **57**, 410–419.

**Decourtye, A., Devillers, J., Genecque, E., Menach, K. Le, Budzinski, H., Cluzeau, S. and Pham-** **Delègue, M. H.** (2005). Comparative Sublethal Toxicity of Nine Pesticides on Olfactory Learning Performances of the Honeybee *Apis mellifera*. *Arch. Environ. Contam. Toxicol.* **48**, 242–250.

**El Hassani, A. K., Dacher, M., Gary, V., Lambin, M., Gauthier, M. and Armengaud, C.** (2008). Effects of Sublethal Doses of Acetamiprid and Thiamethoxam on the Behavior of the Honeybee (*Apis mellifera*). *Arch. Environ. Contam. Toxicol.* **54**, 653–661.

**Frost, E. H., Shutler, D. and Hillier, N. K.** (2013). Effects of fluvalinate on honey bee learning, memory, responsiveness to sucrose, and survival. *J. Exp. Biol.* **216**, 2931–2938.

**Hagen, M. and Dupont, Y. L.** (2013). Inter-tegular span and head width as estimators of fresh and dry body mass in bumblebees (*Bombus* spp.). *Insectes Soc.* **60**, 251–257.

**Han, P., Niu, C.-Y., Lei, C.-L., Cui, J.-J. and Desneux, N.** (2010). Use of an innovative T-tube maze assay and the proboscis extension response assay to assess sublethal effects of GM products and pesticides on learning capacity of the honey bee Apis mellifera L. *Ecotoxicology* **19**, 1612–1619.

**Herbert, L. T., Vazquez, D. E., Arenas, A. and Farina, W. M.** (2014). Effects of field-realistic doses of glyphosate on honeybee appetitive behaviour. *J. Exp. Biol.* **217**, 3457–3464.

**Hesselbach, H. and Scheiner, R.** (2018). Effects of the novel pesticide flupyradifurone (Sivanto) on honeybee taste and cognition. *Sci. Rep.* **8**, 4954.

**Jin, N., Klein, S., Leimig, F., Bischoff, G. and Menzel, R.** (2015). The neonicotinoid clothianidin interferes with navigation of the solitary bee Osmia cornuta in a laboratory test. *J. Exp. Biol.* **218**, 2821–2825.

**Karahan, A., Çakmak, I., Hranitz, J. M., Karaca, I. and Wells, H.** (2015). Sublethal imidacloprid effects on honey bee flower choices when foraging. *Ecotoxicology* **24**, 2017–2025.

**Li, Z., Li, M., Huang, J., Ma, C., Xiao, L., Huang, Q., Zhao, Y., Nie, H. and Su, S.** (2017). Effects of Sublethal Concentrations of Chlorpyrifos on Olfactory Learning and Memory Performances in Two Bee Species, *Apis mellifera* and *Apis cerana*. *Sociobiology* **64**, 174.

**Liao, C., He, X., Wang, Z., Barron, A. B., Zhang, B., Zeng, Z. and Wu, X.** (2018). Short-Term Exposure to Lambda-Cyhalothrin Negatively Affects the Survival and Memory-Related Characteristics of Worker Bees Apis mellifera. *Arch. Environ. Contam. Toxicol.* **75**, 59–65.

**Mamood, A. N. and Waller, G. D.** (1990). Recovery of learning responses by honeybees following a sublethal exposure to permethrin. *Physiol. Entomol.* **15**, 55–60.

**Mengoni Goñalons, C. and Farina, W. M.** (2015). Effects of Sublethal Doses of Imidacloprid on Young Adult Honeybee Behaviour. *PLoS One* **10**, e0140814.

**Mengoni Goñalons, C. and Farina, W. M.** (2018). Impaired associative learning after chronic exposure to pesticides in young adult honey bees. *J. Exp. Biol.* **221**, jeb176644.

**Nakasu, E. Y. T., Williamson, S. M., Edwards, M. G., Fitches, E. C., Gatehouse, J. A., Wright, G. A. and Gatehouse, A. M. R.** (2014). Novel biopesticide based on a spider venom peptide shows no adverse effects on honeybees. *Proc. R. Soc. B Biol. Sci.* **281**, 20140619–20140619.

**Papach, A., Fortini, D., Grateau, S., Aupinel, P. and Richard, F.-J.** (2017). Larval exposure to thiamethoxam and American foulbrood: effects on mortality and cognition in the honey bee Apis mellifera. *J. Apic. Res.* **56**, 475–486.

**Pham-Delegue, M.-H., Girard, C., Metayer, M., Picard-Nizou, A.-L., Hennequet, C., Pons, O. and Jouanin, L.** (2000). Long-term effects of soybean protease inhibitors on digestive enzymes, survival and learning abilities of honeybees. *Entomol. Exp. Appl.* **95**, 21–29.

**Phelps, J. D., Strang, C. G., Gbylik-Sikorska, M., Sniegocki, T., Posyniak, A. and Sherry, D. F.** (2018). Imidacloprid slows the development of preference for rewarding food sources in bumblebees (*Bombus impatiens*). *Ecotoxicology* **27**, 175–187.

**Piiroinen, S. and Goulson, D.** (2016). Chronic neonicotinoid pesticide exposure and parasite stress differentially affects learning in honeybees and bumblebees. *Proc. R. Soc. B Biol. Sci.* **283**, 20160246.

**Piiroinen, S., Botías, C., Nicholls, E. and Goulson, D.** (2016). No effect of low-level chronic neonicotinoid exposure on bumblebee learning and fecundity. *PeerJ* **4**, e1808.

**Ramirez-Romero, R., Chaufaux, J. and Pham-Delègue, M.-H.** (2005). Effects of Cry1Ab protoxin, deltamethrin and imidacloprid on the foraging activity and the learning performances of the honeybee *Apis mellifera* , a comparative approach. *Apidologie* **36**, 601–611.

**Ramirez-Romero, R., Desneux, N., Decourtye, A., Chaffiol, A. and Pham-Delègue, M. H.** (2008). Does Cry1Ab protein affect learning performances of the honey bee *Apis mellifera L.* (Hymenoptera, Apidae)? *Ecotoxicol. Environ. Saf.* **70**, 327–333.

**Rix, R. R. and Christopher Cutler, G.** (2016). Acute Exposure to Worst-Case Concentrations of Amitraz Does Not Affect Honey Bee Learning, Short-Term Memory, or Hemolymph Octopamine Levels. *J. Econ. Entomol.* **110**, tow250.

**Samuelson, E. E. W., Chen-Wishart, Z. P., Gill, R. J. and Leadbeater, E.** (2016). Effect of acute pesticide exposure on bee spatial working memory using an analogue of the radial-arm maze. *Sci. Rep.* **6**, 38957.

**Schneider, S., Eisenhardt, D. and Rademacher, E.** (2012). Sublethal effects of oxalic acid on Apis mellifera (Hymenoptera: Apidae): changes in behaviour and longevity. *Apidologie* **43**, 218–225.

**Stanley, D. A., Smith, K. E. and Raine, N. E.** (2015). Bumblebee learning and memory is impaired by chronic exposure to a neonicotinoid pesticide. *Sci. Rep.* **5**, 16508.

**Stone, J., Abramson, C. and Price, J.** (1997). Task-dependent effects of dicofol (Kelthane) on learning in the honey bee (*Apis mellifera*). *Bull. Environ. Contam. Toxicol.* **58**, 177–183.

**Tan, K., Yang, S., Wang, Z. and Menzel, R.** (2013). Effect of Flumethrin on Survival and Olfactory Learning in Honeybees. *PLoS One* **8**, e66295.

**Tan, K., Chen, W., Dong, S., Liu, X., Wang, Y. and Nieh, J. C.** (2015). A neonicotinoid impairs olfactory learning in Asian honey bees (*Apis cerana*) exposed as larvae or as adults. *Sci. Rep.* **5**, 10989.

**Tan, K., Wang, C., Dong, S., Li, X. and Nieh, J. C.** (2017). The pesticide flupyradifurone impairs olfactory learning in Asian honey bees (*Apis cerana*) exposed as larvae or as adults. *Sci. Rep.* **7**, 17772.

**Taylor, K., Waller, G. and Crowder, L.** (1987). Impairment of a classical conditioned response of the honey bee (*Apis mellifera L.*) by sublethal doses of synthetic pyrethroid insecticides. *Apidologie* **18**, 243–252.

**Thany, S., Bourdin, C., Graton, J., Laurent, A., Mathé-Allainmat, M., Lebreton, J. and Le Questel, J.-Y.** (2015). Similar Comparative Low and High Doses of Deltamethrin and Acetamiprid Differently Impair the Retrieval of the Proboscis Extension Reflex in the Forager Honey Bee (*Apis mellifera*). *Insects* **6**, 805–814.

**Tison, L., Holtz, S., Adeoye, A., Kalkan, Ö., Irmisch, N. S., Lehmann, N. and Menzel, R.** (2017). Effects of sublethal doses of thiacloprid and its formulation Calypso ® on the learning and memory performance of honey bees. *J. Exp. Biol.* **220**, 3695–3705.

**Urlacher, E., Monchanin, C., Rivière, C., Richard, F.-J., Lombardi, C., Michelsen-Heath, S., Hageman, K. J. and Mercer, A. R.** (2016). Measurements of Chlorpyrifos Levels in Forager Bees and Comparison with Levels that Disrupt Honey Bee Odor-Mediated Learning Under Laboratory Conditions. *J. Chem. Ecol.* **42**, 127–138.

**Weick, J. and Thorn, R. S.** (2002). Effects of Acute Sublethal Exposure to Coumaphos or Diazinon on Acquisition and Discrimination of Odor Stimuli in the Honey Bee (Hymenoptera: Apidae). *J. Econ. Entomol.* **95**, 227–236.

**Williamson, S. M. and Wright, G. A.** (2013). Exposure to multiple cholinergic pesticides impairs olfactory learning and memory in honeybees. *J. Exp. Biol.* **216**, 1799–1807.

**Williamson, S. M., Baker, D. D. and Wright, G. A.** (2013). Acute exposure to a sublethal dose of imidacloprid and coumaphos enhances olfactory learning and memory in the honeybee Apis mellifera. *Invertebr. Neurosci.* **13**, 63–70.

**Wright, G. A., Softley, S. and Earnshaw, H.** (2015). Low doses of neonicotinoid pesticides in food rewards impair short-term olfactory memory in foraging-age honeybees. *Sci. Rep.* **5**, 15322.

**Yang, E.-C., Chang, H.-C., Wu, W.-Y. and Chen, Y.-W.** (2012). Impaired Olfactory Associative Behavior of Honeybee Workers Due to Contamination of Imidacloprid in the Larval Stage. *PLoS One* **7**, e49472.

**Zhang, E. and Nieh, J. C.** (2015). The neonicotinoid imidacloprid impairs honey bee aversive learning of simulated predation. *J. Exp. Biol.* **218**, 3199–205.
